# Discovery of benzochalcone derivative as a potential antigastric cancer agent targeting signal transducer and activator of transcription 3 (STAT3)

**DOI:** 10.1080/14756366.2022.2100366

**Published:** 2022-07-18

**Authors:** Jinyun Dong, Jing Yang, Wenkai Yu, Haobin Li, Maohua Cai, Jing-Li Xu, Han-Dong Xu, Yun-Fu Shi, Xiaoqing Guan, Xiang‑Dong Cheng, Jiang‑Jiang Qin

**Affiliations:** a The Cancer Hospital of the University of Chinese Academy of Sciences (Zhejiang Cancer Hospital), Institute of Basic Medicine and Cancer (IBMC), Chinese Academy of Sciences, Hangzhou, China; b Zhejiang Provincial Research Center for Upper Gastrointestinal Tract Cancer, Zhejiang Cancer Hospital, Hangzhou , China; c Zhejiang Key Lab of Prevention, Diagnosis and Therapy of Upper Gastrointestinal Cancer, Zhejiang Cancer Hospital, Hangzhou , China; d School of Pharmaceutical Sciences, Zhejiang Chinese Medical University, Hangzhou, China; e The First Clinical Medical College of Zhejiang, Chinese Medical University, Hangzhou, China

**Keywords:** Benzochalcone analogues, STAT3, gastric cancer

## Abstract

Gastric cancer remains a significant health burden worldwide. In continuation of our previous study and development of effective small molecules against gastric cancer, a series of benzochalcone analogues involving heterocyclic molecules were synthesised and biologically evaluated *in vitro* and *in vivo*. Among them, the quinolin-6-yl substituted derivative KL-6 inhibited the growth of gastric cancer cells (HGC27, MKN28, AZ521, AGS, and MKN1) with a submicromolar to micromolar range of IC_50_, being the most potent one in this series. Additionally, KL-6 significantly inhibited the colony formation, migration and invasion, and effectively induced apoptosis of MKN1 cells in a concentration-dependent manner. The mechanistic study revealed that KL-6 could concentration-dependently suppress STAT3 phosphorylation, which may partly contribute to its anticancer activity. Furthermore, *in vivo* antitumour study on the MKN1 orthotopic tumour model showed that KL-6 effectively inhibited tumour growth (TGI of 78%) and metastasis without obvious toxicity. Collectively, compound KL-6 may support the further development of candidates for gastric cancer treatment.

## Introduction

Cancer has been the second leading cause of death globally, with an estimated 19.3 million new cases and almost 10.0 million cancer deaths in 2020^1^. Even more seriously, a further increase of ∼50% in the cancer burden is predicted between 2020 and 2040^2^. Among all the cancer types, gastric cancer has the characteristics of high metastasis, recurrence, and fatality rate^3^^,^^4^. According to GLOBOCAN 2020 data, gastric carcinoma ranks fifth in morbidity (5.6%) and the fourth (7.7%) in mortality^1^. Therefore, gastric cancer has become a major public health issue worldwide being one of the commonly diagnosed malignant tumours of the digestive system. Currently, surgery combined with chemoradiotherapy is the main treatment for gastric cancer, which could effectively prolong both the progression-free survival and overall survival of patients^5^^,^^6^. Despite upfront responses, drug resistance and recurrence are frequently observed in more than 60% of patients receiving treatment^7^. Even worse, the lack of effective chemotherapeutic drugs for advanced and relapse gastric cancer remain to be serious clinical problems. In this context, the discovery of new oral chemical entities with high efficiency is of great significance for the gastric cancer treatment.

**Figure 1.**
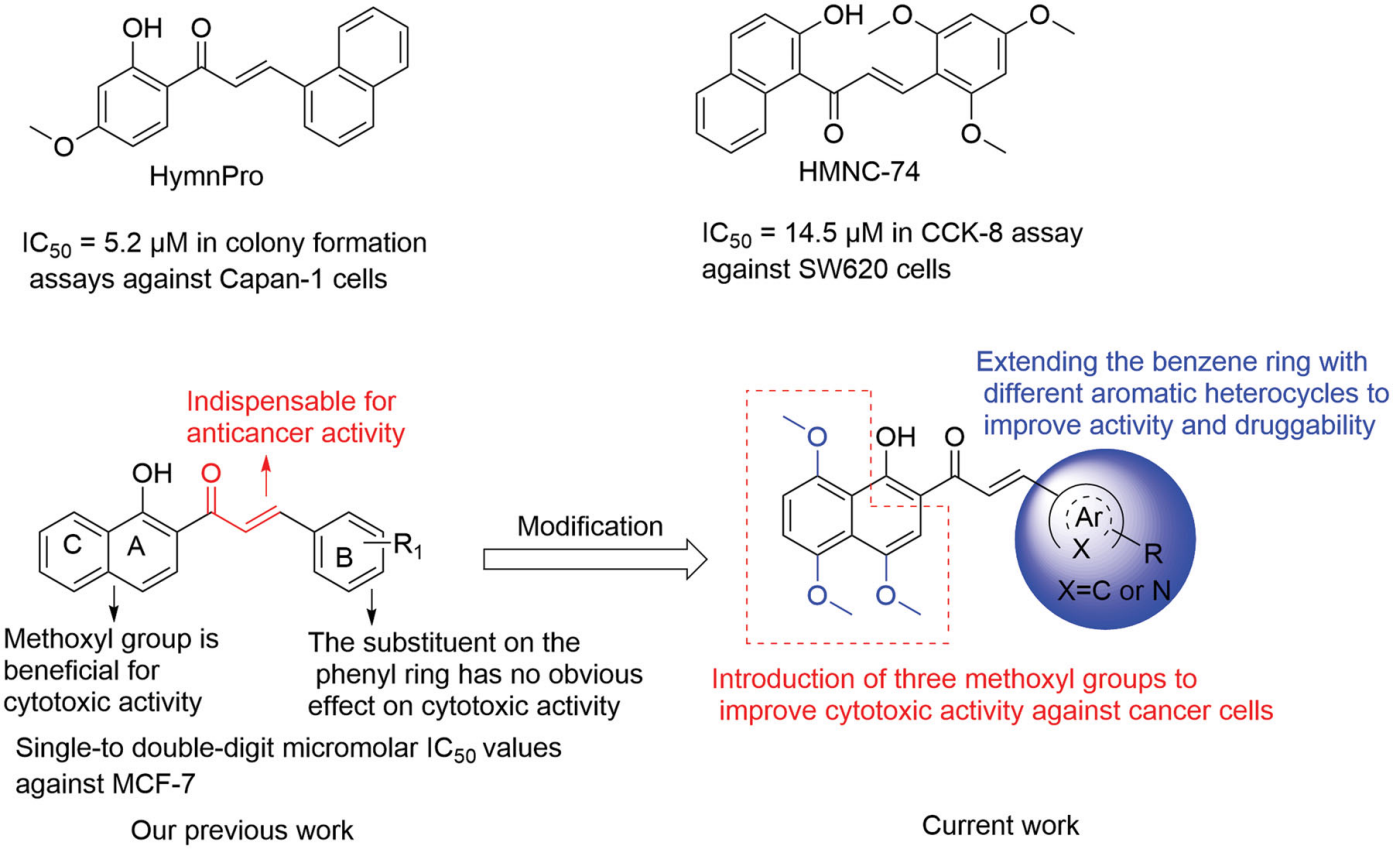
Summary of our previous and current work.

Chalcone (the flavonoid precursor) has long been considered as a privileged scaffold in medicinal chemistry, and its derivatives have attracted considerable research attention for decades due to their broad spectrum of pharmacological activities, including anti-inflammation, antiproliferation, antiangiogenesis, disruption of the cell cycle, and induction of apoptosis^8–10^. Additionally, benzochalcone derivatives (such as HymnPro and HMNC-74, Figure 1) also have aroused the researcher’s interest for their potential application in anticancer field^11^^,^^12^. Similarly, in our previous work, we reported the cytotoxic activity profile of our synthesised benzochalcone derivatives (Figure 1)^13^. Structure-activity relationship (SAR) study suggested that the introduction of methoxyl groups to the naphthalene moiety led to a significant increase in cytotoxic activity against the tested cancer cells, while the substituent on the phenyl ring (B ring) has no obvious effect on activity. Regrettably, no attempt has been made to elucidate the potential target(s) of these chalcones for their anticancer activity.

In continuation of our ongoing research efforts for discovery of new leads with effective cytotoxic activities towards gastric cancer cells, and based on our previous study on chalcones as potential anticancer agents^13^, we herein have designed some benzochalcones by introducing three methoxyl groups to the naphthalene moiety and extending the benzene ring with different heterocyclic molecules. Then we evaluated their inhibitory effects on gastric cancer cells both *in vitro* and *in vivo*. Compared with our previous work, the present study is more comprehensive and systematic in terms of anticancer evaluation. Signal transducer and activator of transcription 3 (STAT3) is a critical transcription factor for many regulatory factors that modulate gene transcription. Accumulated evidence indicates that STAT3 is aberrantly active in various cancer cells, including gastric cancer cell lines (such as HGC27, AGS, MKN1, and MKN28)^14–16^. Thus, STAT3 represents an attractive therapeutic target for the treatment of human cancers. Many synthetic and natural small molecules with various scaffolds have been reported to have STAT3 inhibitory activity. However, very few of them have reached preclinical or clinical studies, and no STAT3 inhibitor has yet been approved for clinical use^17^. Therefore, discovery of new STAT3 inhibitors is still in an urgent demand. Interestingly, chalcone-containing derivatives have been reported to have STAT3 inhibitory activity, as summarised in our previous study^17^. To test our hypothesis regarding whether the anticancer activity of these synthesised benzochalcones was related to STAT3 inhibition, the inhibitory effect of the most potent one (KL-6) in this series on STAT3 was also investigated in this study.

## Experimental

### Materials and methods

#### General procedure for the preparation of 9a–9t

All starting materials were obtained from commercial suppliers and all solvents were used as supplied without further purification unless otherwise indicated. Anhydrous solvents were dried according to standard methods. Proton nuclear magnetic resonance (^1^H NMR) and ^13 ^C NMR were performed on Bruker AVANCE NEO 400 spectrometer in the indicated solvent CDCl_3_. Chemical shifts are expressed in *δ* units (ppm) using tetramethylsilane as an internal standard, and coupling constants (*J*) are expressed in hertz (Hz). Multiplicities are shown as the following abbreviations: s (singlet), brs (broad singlet), d (doublet), t (triplet), and m (multiplet). Reaction process was monitored by thin-layer chromatography (TLC, silica gel GF_254_) and visualised with UV light (*λ* = 254 or 365 nm). Column chromatography was conducted on silica gel (200–300 mesh). HRMS were obtained on Orbitrap Exploris 480.

The intermediates **2**–**8** were prepared from 1,5-dihydroxynaphthalene (**1**) according to our previously reported procedures^9^^,^^18^. To a mixture of 0.5 mmol of 1–(1-hydroxy-4,5,8-trimethoxynaphthalen-2-yl)ethan-1-one (**8**) and different commercially available aromatic (heterocyclic) aldehyde (0.55 mmol) in a 100 ml round bottom flask, 20 ml ethanol, and 1 mmol of KOH were added. The system was heated to reflux until the reaction was complete (indicated by TLC). The reaction mixture was neutralised with saturated ammonium chloride aqueous, and extracted with EtOAc. The combined organic layers were washed with saturated NaCl aqueous, and dried over Na_2_SO_4_. The solvent was removed under reduced pressure and the crude residue was subjected to silica gel column chromatography (40:1–20:1 CH_2_Cl_2_/MeOH) to give title compounds.

##### (E)-1–(1-Hydroxy-4,5,8-trimethoxynaphthalen-2-yl)-3-phenylprop-2-en-1-one (9a)

Reddish-brown solid; M.p. 120 °C–122 °C; yield 39%; ^1^H NMR (400 MHz, CDCl_3_) *δ* 12.81 (s, 1H, -OH), 7.80 (d, *J* = 15.6 Hz, 1H, -CH=CH-), 7.69 (d, *J* = 15.6 Hz, 1H, -CH = CH-), 7.63–7.56 (m, 2H, aromatic H), 7.38–7.33 (m, 2H, aromatic H), 7.30–7.22 (m, 1H, aromatic H), 7.19 (d, *J* = 3.6 Hz, 1H, aromatic H), 6.91 (d, *J* = 8.7 Hz, 1H, aromatic H), 6.80 (d, *J* = 8.7 Hz, 1H, aromatic H), 3.95 (s, 3H, -OCH_3_), 3.87 (s, 3H, -OCH_3_), 3.84 (s, 3H, -OCH_3_); ^13 ^C NMR (101 MHz, CDCl_3_) *δ* 190.93, 155.09, 151.79, 150.19, 147.72, 142.33, 134.17, 129.31, 127.87, 127.51, 123.23, 122.53, 117.50, 116.29, 110.89, 107.06, 106.39, 57.03, 56.76, 55.99; HRMS calcd for [C_22_H_20_O_5_ + H] 365.1389, found 365.1415.

##### (E)-3-([1,1'-Biphenyl]-4-yl)-1–(1-hydroxy-4,5,8-trimethoxynaphthalen-2-yl)prop-2-en-1-one (9b)

Reddish-brown solid; M.p. 179 °C–181 °C; yield 32%; *δ* ^1^H NMR (400 MHz, CDCl_3_) *δ* 12.86 (s, 1H, -OH), 7.84 (d, *J* = 15.6 Hz, 1H, -CH=CH-), 7.72 (d, *J* = 15.6 Hz, 1H, -CH = CH-), 7.67 (d, *J* = 7.9 Hz, 2H, aromatic H), 7.61–7.53 (m, 4H, aromatic H), 7.39 (t, *J* = 7.5 Hz, 2H, aromatic H), 7.31 (t, *J* = 7.3 Hz, 1H, aromatic H), 7.22 (s, 1H, aromatic H), 6.92 (d, *J* = 8.7 Hz, 1H, aromatic H), 6.83 (d, 1H, aromatic H), 3.97 (s, 3H, -OCH_3_), 3.88 (s, 3H, -OCH_3_), 3.85 (s, 3H, -OCH_3_); ^13 ^C NMR (101 MHz, CDCl_3_) *δ* 190.85, 155.18, 151.84, 150.20, 147.73, 142.04, 141.90, 139.19, 133.17, 131.81, 128.04, 127.88, 126.52, 126.03, 123.05, 122.57, 117.54, 116.31, 110.92, 107.14, 106.41, 57.08, 56.78, 56.01; HRMS calcd for [C_28_H_24_O_5_ + H] 441.1702, found 441.1736.

##### (E)-1–(1-Hydroxy-4,5,8-trimethoxynaphthalen-2-yl)-3–(4-phenoxyphenyl)prop-2-en-1-one (9c)

Reddish-brown solid; M.p. 158 °C–160 °C; yield 33%; ^1^H NMR (400 MHz, CDCl_3_) *δ* 13.00 (s, 1H, -OH), 7.85 (d, *J* = 15.6 Hz, 1H, -CH=CH-), 7.67 (d, *J* = 13.8 Hz, 1H, -CH = CH-), 7.64 (d, *J* = 6.8 Hz, 1H, aromatic H), 7.40–7.35 (m, 2H, aromatic H), 7.27 (s, 1H), 7.20–7.14 (m, 1H, aromatic H), 7.09–7.05 (m, 2H, aromatic H), 7.03–6.97 (m, 4H, aromatic H), 6.88 (d, *J* = 8.8 Hz, 1H, aromatic H), 4.03 (s, 3H, -OCH_3_), 3.94 (s, 3H, -OCH_3_), 3.92 (s, 3H, -OCH_3_); ^13 ^C NMR (101 MHz, CDCl_3_) *δ* 190.84, 158.57, 155.06, 151.80, 150.12, 147.66, 141.77, 129.25, 128.91, 128.70, 127.61, 123.09, 121.80, 118.65, 118.40, 117.87, 117.76, 117.38, 110.88, 107.10, 106.39, 57.05, 56.73, 55.96; HRMS calcd for [C_28_H_24_O_6_ + H] 457.1651, found 457.1686.

##### (E)-1–(1-Hydroxy-4,5,8-trimethoxynaphthalen-2-yl)-3-(naphthalen-2-yl)prop-2-en-1-one (9d)

Reddish-brown solid; M.p. 176 °C–178 °C; yield 36%; ^1^H NMR (400 MHz, CDCl_3_) *δ* 12.93 (s, 1H, -OH), 8.10–7.98 (m, 2H, -CH=CH-), 7.92–7.89 (m, 1H, aromatic H), 7.87–7.81 (m, 4H, aromatic H), 7.55–7.49 (m, 2H, aromatic H), 7.32 (s, 1H, aromatic H), 7.00 (d, *J* = 8.8 Hz, 1H, aromatic H), 6.89 (d, *J* = 8.7 Hz, 1H, aromatic H), 4.05 (s, 3H, -OCH_3_), 3.97 (s, 3H, -OCH_3_), 3.91 (s, 3H, -OCH_3_); ^13 ^C NMR (101 MHz, CDCl_3_) *δ* 190.74, 155.16, 151.74, 150.13, 147.70, 142.36, 133.23, 132.31, 131.64, 129.55, 127.58, 127.56, 126.72, 126.19, 125.61, 123.27, 122.82, 122.50, 117.46, 116.28, 110.85, 107.07, 106.32, 57.03, 56.69, 55.91; HRMS calcd for [C_26_H_22_O_5_ + H] 415.1545, found 415.1579.

##### (E)-1–(1-Hydroxy-4,5,8-trimethoxynaphthalen-2-yl)-3–(4-morpholinophenyl)prop-2-en-1-one (9e)

Reddish-brown solid; M.p. 166 °C–168 °C; yield 31%; ^1^H NMR (400 MHz, CDCl_3_) *δ* 13.57 (s, 1H, -OH), 7.86 (d, *J* = 15.4 Hz, 1H, -CH=CH-), 7.65–7.54 (m, 3H, -CH = CH-, aromatic H), 7.28 (s, 1H, aromatic H), 7.02–6.98 (m, 1H, aromatic H), 6.92–6.89 (m, 2H, aromatic H), 6.89–6.86 (m, 1H, aromatic H), 4.02 (s, 3H, -OCH_3_), 3.94 (s, 3H, -OCH_3_), 3.92 (s, 3H, -OCH_3_), 3.88–3.85 (m, 4H, -O(CH_2_CH_2_)_2_N-), 3.31–3.25 (m, 4H, -O(CH_2_CH_2_)_2_N-); ^13 ^C NMR (101 MHz, CDCl_3_) *δ* 191.15, 156.10, 152.15, 149.96, 147.42, 143.10, 131.70, 129.27, 127.72, 126.62, 125.08, 122.70, 118.84, 117.79, 115.56, 113.64, 111.07, 107.49, 106.53, 65.60, 57.40, 56.80, 56.04, 47.00; HRMS calcd for [C_26_H_27_NO_6_ + H] 450.1917, found 450.1950.

##### (E)-1–(1-Hydroxy-4,5,8-trimethoxynaphthalen-2-yl)-3–(3-morpholinophenyl)prop-2-en-1-one (9f)

Reddish-brown solid; M.p. 135 °C–137 °C; yield 30%; ^1^H NMR (400 MHz, CDCl_3_) *δ* 12.85 (s, 1H, -OH), 7.82 (d, *J* = 15.6 Hz, 1H, -CH=CH-), 7.70 (d, *J* = 15.6 Hz, 1H, -CH = CH-), 7.32 (t, *J* = 7.9 Hz, 1H, aromatic H), 7.25–7.21 (m, 1H, aromatic H), 7.16 (s, 1H, aromatic H), 7.00–6.96 (m, 2H, aromatic H), 6.92–6.84 (m, 2H, aromatic H), 4.03 (s, 3H, -OCH_3_), 3.93 (s, 3H, -OCH_3_), 3.92 (s, 3H, -OCH_3_), 3.90–3.86 (m, 4H, -O(CH_2_CH_2_)_2_N-), 3.22–3.19 (m, 4H, -O(CH_2_CH_2_)_2_N-); ^13 ^C NMR (101 MHz, CDCl_3_) *δ* 191.05, 155.02, 151.79, 150.17, 147.71, 142.91, 135.12, 128.64, 128.42, 123.30, 122.54, 118.98, 117.50, 116.79, 116.34, 115.15, 110.84, 107.36, 106.38, 65.78, 57.19, 56.75, 56.00, 48.30; HRMS calcd for [C_26_H_27_NO_6_ + H] 450.1917, found 450.1953.

##### (E)-1–(1-Hydroxy-4,5,8-trimethoxynaphthalen-2-yl)-3–(2-morpholinophenyl)prop-2-en-1-one (9g)

Reddish-brown solid; M.p. 157 °C–159 °C; yield 28%; ^1^H NMR (400 MHz, CDCl_3_) *δ* 12.89 (s, 1H, -OH), 8.23 (d, *J* = 15.8 Hz, 1H, -CH=CH-), 7.78–7.68 (m, 2H, -CH = CH-, aromatic H), 7.44–7.35 (m, 1H, aromatic H), 7.27 (s, 1H, aromatic H), 7.12 (t, *J* = 7.6 Hz, 1H, aromatic H), 7.08 (dd, *J* = 8.2, 1.1 Hz, 1H, aromatic H), 6.99 (d, *J* = 8.6 Hz, 1H, aromatic H), 6.89 (d, *J* = 8.8 Hz, 1H, aromatic H), 4.03 (s, 3H, -OCH_3_), 3.94 (s, 3H, -OCH_3_), 3.92 (s, 3H, -OCH_3_), 3.91–3.87 (m, 4H, -O(CH_2_CH_2_)_2_N-), 3.03–2.95 (m, 4H, -O(CH_2_CH_2_)_2_N-); ^13 ^C NMR (101 MHz, CDCl_3_) *δ* 191.39, 154.88, 151.79, 150.22, 147.72, 139.90, 137.87, 131.74, 130.05, 128.36, 127.40, 123.19, 122.44, 122.13, 117.77, 117.56, 110.78, 107.01, 106.46, 66.12, 57.00, 56.77, 56.04, 52.18; HRMS calcd for [C_26_H_27_NO_6_ + H] 450.1917, found 450.1952.

##### (E)-1–(1-Hydroxy-4,5,8-trimethoxynaphthalen-2-yl)-3–(2-(4-methylpiperazin-1-yl)phenyl)prop-2-en-1-one (9h)

Reddish-brown solid; M.p. 143 °C–145 °C; yield 23%; ^1^H NMR (400 MHz, CDCl_3_) *δ* 12.72 (s, 1H, -OH), 8.17 (d, *J* = 15.7 Hz, 1H, -CH=CH-), 7.75–7.67 (m, 2H, -CH = CH-, aromatic H), 7.42–7.29 (m, 2H, aromatic H), 7.11–7.06 (m, 2H, aromatic H), 6.97 (d, *J* = 9.1 Hz, 1H, aromatic H), 6.87 (d, *J* = 8.7 Hz, 1H, aromatic H), 4.02 (s, 3H, -OCH_3_), 3.93 (s, 3H, -OCH_3_), 3.91 (s, 3H, -OCH_3_), 3.19–3.01 (m, 4H, -N(CH_2_CH_2_)_2_N-), 2.85 (brs, 4H, -N(CH_2_CH_2_)_2_N-), 2.49 (s, 3H, -NCH_3_); ^13 ^C NMR (101 MHz, CDCl_3_) *δ* 191.38, 151.66, 151.21, 150.27, 147.79, 144.03, 139.55, 130.10, 128.34, 127.16, 123.51, 122.40, 118.17, 117.49, 116.57, 110.65, 107.02, 106.61, 106.41, 56.97, 56.73, 56.04, 54.02, 53.74, 50.72, 50.64, 44.11; HRMS calcd for [C_27_H_30_N_2_O_5_ + H] 463.2233, found 463.2268.

##### (E)-3–(4-(1,1-Dioxidothiomorpholino)phenyl)-1–(1-hydroxy-4,5,8-trimethoxynaphthalen-2-yl)prop-2-en-1-one (9i)

Reddish-brown solid; M.p. 177 °C–178 °C; yield 25%; ^1^H NMR (400 MHz, CDCl_3_) *δ* 13.18 (s, 1H, -OH), 7.82 (d, *J* = 15.5 Hz, 1H, -CH=CH-), 7.63 (brs, 1H, -CH = CH-), 7.60 (d, *J* = 7.2 Hz, 1H, aromatic H), 7.26 (s, 1H, aromatic H), 6.99 (d, *J* = 8.8 Hz, 1H, aromatic H), 6.95–6.85 (m, 4H, aromatic H), 4.03 (s, 3H, -OCH_3_), 3.98–3.94 (m, 4H, -SO_2_(CH_2_CH_2_)_2_N-), 3.93 (s, 3H, -OCH_3_), 3.92 (s, 3H, -OCH_3_), 3.13–3.10 (m, 4H,-SO_2_(CH_2_CH_2_)_2_N-); ^13 ^C NMR (101 MHz, CDCl_3_) *δ* 190.93, 155.50, 151.93, 150.08, 147.59, 147.50, 141.99, 130.61, 128.10, 126.17, 122.59, 120.95, 117.62, 116.00, 114.67, 114.30, 110.92, 107.43, 106.47, 57.30, 56.76, 56.02, 49.41, 45.75; HRMS calcd for [C_26_H_27_NO_7_S + H] 498.1586, found 498.1624.

##### (E)-1–(1-Hydroxy-4,5,8-trimethoxynaphthalen-2-yl)-3–(4-(pyridin-4-yl)phenyl)prop -2-en-1-one (9j)

Reddish-brown solid; M.p. 182 °C–184 °C; yield 30%; ^1^H NMR (400 MHz, CDCl_3_) *δ* 12.60 (s, 1H, -OH), 8.69–8.64 (m, 2H, aromatic H (pyridyl)), 7.84 (m, 2H, -CH=CH-), 7.76 (d, *J* = 8.4 Hz, 2H, aromatic H), 7.67 (d, *J* = 8.3 Hz, 2H, aromatic H), 7.54–7.51 (m, 2H, aromatic H), 7.24 (s, 1H, aromatic H), 6.97 (d, *J* = 8.8 Hz, 1H, aromatic H), 6.87 (d, *J* = 8.7 Hz, 1H, aromatic H), 4.02 (s, 3H, -OCH_3_), 3.93 (s, 3H, -OCH_3_), 3.90 (s, 3H, -OCH_3_); ^13 ^C NMR (101 MHz, CDCl_3_) *δ* 190.52, 154.67, 151.63, 150.31, 149.11, 147.88, 146.57, 140.86, 138.49, 135.12, 131.12, 128.20, 126.45, 124.53, 122.47, 120.48, 117.40, 110.77, 107.06, 106.35, 56.98, 56.74, 56.00; HRMS calcd for [C_27_H_23_NO_5_ + H] 442.1654, found 442.1693.

##### (E)-1–(1-Hydroxy-4,5,8-trimethoxynaphthalen-2-yl)-3–(3-(pyridin-4-yl)phenyl)prop-2-en-1-one (9k)

Reddish-brown solid; M.p. 181 °C–183 °C; yield 27%; ^1^H NMR (400 MHz, CDCl_3_) *δ* 12.48 (s, 1H, -OH), 8.71–8.67 (m, 2H, aromatic H (pyridyl)), 7.89–7.84 (m, 2H, -CH=CH-), 7.75–7.71 (m, 1H, aromatic H), 7.65–7.61 (m, 1H, aromatic H), 7.58–7.54 (m, 2H, aromatic H), 7.54–7.50 (m, 2H, aromatic H), 7.27 (s, 1H, aromatic H), 6.97 (d, *J* = 8.8 Hz, 1H, aromatic H), 6.87 (d, *J* = 8.7 Hz, 1H, aromatic H), 4.03 (s, 3H, -OCH_3_), 3.93 (s, 3H, -OCH_3_), 3.91 (s, 3H, -OCH_3_); ^13 ^C NMR (101 MHz, CDCl_3_) *δ* 190.51, 154.36, 151.52, 150.31, 149.06, 147.91, 147.02, 141.12, 137.79, 135.23, 128.68, 127.86, 127.69, 126.10, 124.72, 122.41, 120.78, 117.33, 116.84, 110.64, 107.16, 106.27, 56.99, 56.70, 55.97, HRMS calcd for [C_27_H_23_NO_5_ + H] 442.1654, found 442.1687.

##### (E)-1–(1-Hydroxy-4,5,8-trimethoxynaphthalen-2-yl)-3–(2-(pyridin-4-yl)phenyl) prop-2-en-1-one (9l)

Reddish-brown solid; M.p. 172 °C–174 °C; yield 33%; ^1^H NMR (400 MHz, CDCl_3_) *δ* 12.40 (s, 1H, -OH), 8.71–8.65 (m, 2H, aromatic H (pyridyl)), 7.92–7.85 (m, 1H, -CH=CH-) , 7.82–7.68 (m, 2H, -CH = CH-, aromatic H), 7.52–7.45 (m, 2H, aromatic H), 7.37 (dd, *J* = 5.7, 3.3 Hz, 1H, aromatic H), 7.34–7.30 (m, 2H, aromatic H), 7.21 (s, 1H, aromatic H), 6.97 (d, *J* = 8.7 Hz, 1H, aromatic H), 6.87 (d, *J* = 8.7 Hz, 1H, aromatic H), 4.04 (s, 3H, -OCH_3_), 3.92 (s, 3H, -OCH_3_), 3.91 (s, 3H, -OCH_3_); ^13 ^C NMR (101 MHz, CDCl_3_) *δ* 190.26, 154.33, 151.54, 150.34, 148.70, 147.87, 147.03, 139.72, 139.20, 132.46, 129.17, 128.99, 127.83, 126.61, 126.10, 123.72, 122.36, 117.34, 116.70, 110.69, 106.83, 106.34, 56.82, 56.73, 56.01; HRMS calcd for [C_27_H_23_NO_5_ + H] 442.1654, found 442.1687.

##### (E)-1–(1-Hydroxy-4,5,8-trimethoxynaphthalen-2-yl)-3-(pyridin-2-yl)prop-2-en-1-one (9m)

Reddish-brown solid; M.p. 162 °C–164 °C; yield 24%; ^1^H NMR (400 MHz, CDCl_3_) *δ* 12.23 (s, 1H, -OH), 8.87 (d, *J* = 2.2 Hz, 1H, aromatic H), 8.60 (dd, *J* = 4.8, 1.7 Hz, 1H, aromatic H), 7.96 (dt, *J* = 7.9, 2.0 Hz, 1H, aromatic H), 7.88 (d, *J* = 15.7 Hz, 1H, -CH=CH-), 7.79 (d, *J* = 15.8 Hz, 1H, -CH = CH-), 7.34 (ddd, *J* = 7.9, 4.8, 0.9 Hz, 1H, aromatic H), 7.26 (s, 1H, aromatic H), 6.97 (d, *J* = 8.7 Hz, 1H, aromatic H), 6.88 (d, *J* = 8.8 Hz, 1H, aromatic H), 4.04 (s, 3H, -OCH_3_), 3.94 (s, 3H, -OCH_3_), 3.91 (s, 3H, -OCH_3_); ^13 ^C NMR (101 MHz, CDCl_3_) *δ* 189.93, 153.99, 151.39, 150.45, 149.60, 148.86, 148.04, 137.65, 133.74, 130.18, 126.17, 122.73, 122.34, 117.24, 117.01, 110.62, 106.77, 106.26, 56.75, 56.72, 55.98; HRMS calcd for [C_21_H_19_NO_5_ + H] 366.1341, found 366.1372.

##### (E)-1–(1-Hydroxy-4,5,8-trimethoxynaphthalen-2-yl)-3–(5-methoxypyridin-2-yl) prop-2-en-1-one (9n)

Reddish-brown solid; M.p. 127 °C–129 °C; yield 28%; ^1^H NMR (400 MHz, CDCl_3_) *δ* 13.46 (s, 1H, -OH), 8.41 (d, *J* = 2.9 Hz, 1H, aromatic H), 8.16 (d, *J* = 15.1 Hz, 1H, -CH=CH-), 7.82 (d, *J* = 15.1 Hz, 1H, -CH = CH-), 7.46 (d, *J* = 8.6 Hz, 1H, aromatic H), 7.35 (s, 1H, aromatic H), 7.24–7.17 (m, 1H, aromatic H), 7.01 (d, *J* = 8.7 Hz, 1H, aromatic H), 6.88 (d, *J* = 8.7 Hz, 1H, aromatic H), 4.02 (s, 3H, -OCH_3_), 3.95 (s, 3H, -OCH_3_), 3.91 (s, 3H, -OCH_3_), 3.91 (s, 3H, -OCH_3_); ^13 ^C NMR (101 MHz, CDCl_3_) *δ* 191.16, 156.50, 155.32, 152.20, 150.05, 147.59, 144.86, 140.17, 137.76, 125.47, 124.18, 122.89, 119.02, 117.66, 115.58, 111.49, 106.91, 106.56, 57.13, 56.88, 56.00, 54.73; HRMS calcd for [C_22_H_21_NO_6_ + H] 396.1447, found 396.1474.

##### (E)-1–(1-Hydroxy-4,5,8-trimethoxynaphthalen-2-yl)-3-(quinolin-6-yl)prop-2-en-1-one (9o)

Reddish-brown solid; M.p. 186 °C–188 °C; yield 39%; ^1^H NMR (400 MHz, CDCl_3_) *δ* 12.52 (s, 1H, -OH), 8.99–8.83 (m, 1H, aromatic H), 8.26 (d, *J* = 12 Hz, 1H, -CH=CH-), 8.19 (d, *J* = 12 Hz, 1H, -CH = CH-), 8.11 (dd, *J* = 8.8, 1.8 Hz, 1H, aromatic H), 8.04 (s, 1H, aromatic H), 8.01–7.89 (m, 2H, aromatic H), 7.48 (dd, *J* = 8.3, 4.3 Hz, 1H, aromatic H), 7.31 (s, 1H, aromatic H), 6.99 (d, *J* = 3.5 Hz, 1H, aromatic H), 6.90 (d, *J* = 8.8 Hz, 1H, aromatic H), 4.06 (s, 3H, -OCH_3_), 3.97 (s, 3H, -OCH_3_), 3.93 (s, 3H, -OCH_3_). ^13 ^C NMR (101 MHz, CDCl_3_) *δ* 190.35, 154.54, 151.58, 150.33, 149.98, 147.92, 147.75, 140.78, 135.76, 132.66, 128.88, 128.85, 127.36, 126.70, 125.06, 122.44, 120.79, 117.36, 116.79, 110.75, 106.95, 106.31, 56.91, 56.73, 55.99; HRMS calcd for [C_25_H_21_NO_5_ + H] 416.1498, found 416.1526.

##### (E)-1–(1-Hydroxy-4,5,8-trimethoxynaphthalen-2-yl)-3-(quinolin-8-yl)prop-2-en-1-one (9p)

Reddish-brown solid; M.p. 167 °C–169 °C; yield 30%; ^1^H NMR (400 MHz, CDCl_3_) *δ* 13.46 (s, 1H, -OH), 9.18–8.87 (m, 2H, aromatic H), 8.24–8.10 (m, 3H, aromatic H, -CH=CH-), 7.88 (dd, *J* = 8.1, 1.3 Hz, 1H, aromatic H), 7.60 (t, *J* = 7.7 Hz, 1H, aromatic H), 7.47 (dd, *J* = 8.2, 4.2 Hz, 1H, aromatic H), 7.35 (s, 1H, aromatic H), 7.00 (d, *J* = 8.8 Hz, 1H, aromatic H), 6.88 (d, *J* = 8.8 Hz, 1H, aromatic H), 4.02 (s, 3H, -OCH_3_), 3.96 (s, 3H, -OCH_3_), 3.92 (s, 3H, -OCH_3_); ^13 ^C NMR (101 MHz, CDCl_3_) *δ* 191.63, 156.17, 152.18, 150.03, 149.22, 147.49, 145.47, 139.28, 135.42, 132.73, 129.16, 127.83, 127.52, 125.37, 125.33, 122.74, 120.57, 117.73, 115.60, 111.29, 107.19, 106.55, 57.06, 56.86, 56.03; HRMS calcd for [C_25_H_21_NO_5_ + H] 416.1498, found 416.1527.

##### (E)-1–(1-Hydroxy-4,5,8-trimethoxynaphthalen-2-yl)-3-(quinolin-7-yl)prop-2-en-1-one (9q)

Reddish-brown solid; M.p. 173 °C–175 °C; yield 24%; ^1^H NMR (400 MHz, CDCl_3_) *δ* 12.63 (s, 1H, -OH), 8.95 (dd, *J* = 4.2, 1.7 Hz, 1H, aromatic H), 8.39 (s, 1H, aromatic H,), 8.20 − 8.15 (m, 1H, -CH=CH-), 8.02 (d, *J* = 3.3 Hz, 2H, -CH = CH-, aromatic H), 7.85 (d, *J* = 1.2 Hz, 2H, aromatic H), 7.43 (dd, *J* = 8.3, 4.2 Hz, 1H, aromatic H), 7.31 (s, 1H, aromatic H), 7.00 (d, *J* = 8.7 Hz, 1H, aromatic H), 6.90 (d, *J* = 8.7 Hz, 1H, aromatic H), 4.06 (s, 3H, -OCH_3_), 3.97 (s, 3H, -OCH_3_), 3.93 (s, 3H, -OCH_3_); ^13 ^C NMR (101 MHz, CDCl_3_) *δ* 190.40, 154.79, 151.69, 150.35, 150.16, 147.91, 147.28, 141.02, 135.52, 134.83, 129.11, 128.23, 127.39, 125.46, 124.68, 122.52, 120.82, 117.43, 116.68, 110.91, 106.80, 106.36, 56.88, 56.80, 56.01; HRMS calcd for [C_25_H_21_NO_5_ + H] 416.1498, found 416.1527.

##### (E)-1–(1-Hydroxy-4,5,8-trimethoxynaphthalen-2-yl)-3-(quinolin-3-yl)prop-2-en-1-one (9r)

Reddish-brown solid; M.p. 90 °C–92 °C; yield 20%; ^1^H NMR (400 MHz, CDCl_3_) *δ* 12.29 (s, 1H, -OH), 9.24 (d, *J* = 2.2 Hz, 1H, aromatic H), 8.36 (d, *J* = 2.1 Hz, 1H, aromatic H), 8.20 − 8.11 (m, 1H, -CH=CH-), 8.10–7.92 (m, 2H, -CH = CH-, aromatic H), 7.89 (dd, *J* = 8.2, 1.4 Hz, 1H, aromatic H), 7.80–7.73 (m, 1H, aromatic H), 7.60 (ddd, *J* = 8.1, 6.9, 1.2 Hz, 1H, aromatic H), 7.30 (s, 1H, aromatic H), 6.99 (d, *J* = 8.7 Hz, 1H, aromatic H), 6.90 (d, *J* = 8.8 Hz, 1H, aromatic H), 4.07 (s, 3H, -OCH_3_), 3.96 (s, 3H, -OCH_3_), 3.93 (s, 3H, -OCH_3_); ^13 ^C NMR (101 MHz, CDCl_3_) *δ* 189.93, 154.09, 151.46, 150.48, 148.42, 148.08, 147.15, 137.90, 135.07, 129.60, 128.17, 127.45, 127.36, 126.82, 126.45, 126.05, 122.42, 117.30, 117.10, 110.72, 106.87, 106.30, 56.81, 56.77, 56.03; HRMS calcd for [C_25_H_21_NO_5_ + H] 416.1498, found 416.1530.

##### (E)-1–(1-Hydroxy-4,5,8-trimethoxynaphthalen-2-yl)-3-(quinolin-5-yl)prop-2-en-1-one (9s)

Reddish-brown solid; M.p. 107 °C–109 °C; yield 27%; ^1^H NMR (400 MHz, CDCl_3_) *δ* 12.44 (s, 1H, -OH), 8.99 (d, *J* = 4.4 Hz, 1H, aromatic H), 8.71 (dd, *J* = 8.6, 1.4 Hz, 1H, aromatic H), 8.58 (d, *J* = 15.3 Hz, 1H, aromatic H), 8.25–8.15 (m, 1H, -CH=CH-), 8.05–7.99 (m, 1H, -CH = CH-), 7.92 (d, *J* = 15.4 Hz, 1H, aromatic H), 7.83–7.77 (m, 1H, aromatic H), 7.57–7.52 (m, 1H, aromatic H), 7.31 (s, 1H, aromatic H), 7.00 (d, *J* = 8.7 Hz, 1H, aromatic H), 6.90 (d, *J* = 8.8 Hz, 1H, aromatic H), 4.05 (s, 3H, -OCH_3_), 3.96 (s, 3H, -OCH_3_), 3.93 (s, 3H, -OCH_3_); ^13 ^C NMR (101 MHz, CDCl_3_) *δ* 190.15, 151.92, 151.53, 150.42, 149.18, 148.02, 141.92, 136.57, 132.20, 131.75, 130.08, 128.40, 127.65, 126.10, 124.60, 122.46, 120.56, 117.34, 116.88, 110.77, 106.84, 106.34, 56.84, 56.76, 56.01; HRMS calcd for [C_25_H_21_NO_5_ + H] 416.1498, found 416.1525.

##### (E)-1–(1-Hydroxy-4,5,8-trimethoxynaphthalen-2-yl)-3-(isoquinolin-5-yl)prop-2-en-1-one (9t)

Reddish-brown solid; M.p. 177 °C–179 °C; yield 24%; ^1^H NMR (400 MHz, CDCl_3_) *δ* 12.22 (s, 1H, -OH), 9.37 (s, 1H, aromatic H), 8.63 (d, *J* = 6.1 Hz, 1H, aromatic H), 8.52 (d, *J* = 15.5 Hz, 1H, -CH=CH-), 8.29–8.17 (m, 2H, aromatic H), 8.11 (d, *J* = 8.3 Hz, 1H, aromatic H), 7.95 (d, *J* = 15.4 Hz, 1H, -CH = CH-), 7.75 (t, *J* = 7.8 Hz, 1H, aromatic H), 7.32 (s, 1H, aromatic H), 7.00 (d, *J* = 8.7 Hz, 1H, aromatic H), 6.90 (d, *J* = 8.7 Hz, 1H, aromatic H), 4.06 (s, 3H, -OCH_3_), 3.96 (s, 3H, -OCH_3_), 3.93 (s, 3H, -OCH_3_); ^13 ^C NMR (101 MHz, CDCl_3_) *δ* 191.19, 154.43, 151.41, 150.56, 148.16, 141.31, 135.64, 134.20, 134.09, 130.83, 129.25, 129.10, 128.58, 126.85, 122.86, 117.92, 117.28, 116.76, 116.75, 110.71, 106.91, 106.35, 56.81, 56.77, 56.05; HRMS calcd for [C_25_H_21_NO_5_ + H] 416.1498, found 416.1528.

#### Culture of human gastric cancer cell lines

Human gastric cancer cell lines, including HGC27, MKN28, AZ521, AGS, and MKN1 were obtained from the Cell Bank of the Chinese Academy of Science (Shanghai, China). All the cell lines were maintained in RPMI1640 (Hyclone) supplemented with 10% foetal bovine serum and 1% penicillin/streptomycin (Northend Biotechnology, Hangzhou, China). All of the cells were incubated with a humidified air of 5% CO_2_ at 37 °C.

#### Cell viability assay

The tested compounds were prepared to the stock concentration of 10 mM with dimethyl sulfoxide (DMSO) and stored at −20 °C. Gastric cancer cell lines HGC27, MKN28, AZ521, AGS, and MKN1 were cultured in 96-well plates overnight and then treated with DMSO or compounds **9a–t** or positive controls at various concentrations (0.1–100 μM). After 72 h, Cell Counting Kit 8 (CCK-8, Biosharp, Hefei, China) assay was performed to examine the effects of the tested compounds on gastric cancer cell viability, and the absorbance was measured at 450 nm. The IC_50_ values were calculated by GraphPad Prism 5 software (GraphPad Software Inc, San Diego, USA). For preliminary evaluation, the experiment was conducted one time with three replicates of each compound (except KL-6, which was tested for three times).

#### Colony formation assay

A colony formation assay was employed to study the ability of cells to grow into a colony as described previously with minor modification^19^. Briefly, 5 × 10^2^ of MKN1 cells were seeded in each well of 6-well plate overnight and then treated with DMSO or compound KL-6 (0, 1, 2, and 5 μM) for 24 h and then refreshed the medium, cultured for another 15 days until the clone can be observed with naked eyes. Then, after washing by cold PBS two times, the cells were fixed by 4% paraformaldehyde and stained by 2.5% crystal violet. Images were acquired by a scanner and counted by the naked eye. The experiment was independently performed three times.

#### Wound healing assay

A wound healing assay was performed as previously described with minor modification^20^. Briefly, cells were seeded in a 12-well plate and incubated until the cell density reached about 90% during the wounding healing assay. The cell surface was scratched by a 10 μl pipet tip and washed with PBS for two times. Then, fresh medium involving compound KL-6 at gradient concentrations (0, 1, and 2 μM) was added to the plate followed by incubating for a further 12 h or 24 h. The wound scratched was photographed by an inverted microscope at the time of 0, 12, and 24 h. The experiment was independently repeated three times.

#### Transwell assay

A transwell assay was performed as previously described with minor modification^21^. Briefly, spread matrigel was added to the upper compartment of the transwell chamber (Biosharp, China) and incubate for 6 h in a 37 °C incubator. And then 8 × 10^4^ cells suspended in 200 μl serum-free cell suspension were plated in an upper chamber, following by adding 700 μl medium containing 20% foetal bovine serum and KL-6 at gradient concentration (0, 1, and 2 μM) in the lower chamber. After culturing for 48 h, it was fixed with 4% paraformaldehyde and stained with 2.5% crystal violet. The cells in the upper chamber wall were swabbed with cotton swabs. At last, the cells were washed until the staining was clear enough and photographed by an inverted microscope and quantified by manual counting. The experiment was independently conducted in triplicate.

#### Cell apoptosis assay

Cell apoptosis assay was measured by flow cytometer as described previously^22^. Briefly, MKN1 cells were seeded in 6-well plates (6 × 10^5^ cells/well) and allowed to attach overnight, and the cells were treated with compound KL-6 at various concentrations (0, 1, 2, or 5 µM), followed by another 48 h incubation. The cells were harvested and washed with pre-cooling PBS. The binding buffer and staining reagents of FITC Annexin V Apoptosis Detection Kit I (BD Pharmingen, USA) were added to re-suspend the cells. Each sample was detected by flow cytometer (BD Biosciences, CA, USA). The experiment was independently replicated three times.

#### In vivo studies

All animal studies were carried out according to the Guide for the Care and Use of Laboratory Animal Resources, and were approved by Zhejiang Chinese Medical University Laboratory Animal Research Centre (No. 202105–0186). Nude mice were subcutaneous injection of MKN1 cells for 4 weeks, and then executed mice to separate tumour block (1 mm^3^ tumour), which was transplanted to the stomach corner with medical BioGlue. The mice bearing MKN1 orthotopic tumours divided into two groups (control group and test group. KL-6 was dissolved in PEG400:ethanol:saline (4:1:2), which was applied to the treatment group via intraperitoneal injection at a dose of 5 mg/kg once a day for a period of 28 days. The size of the tumour *in vivo* and the weight of mice were measured and compared with that of the xenograft tumours in mice of the placebo control. d-luciferin (30 mg/ml) was injected with the mice 10 min before BLI imaging.

The animals were then anaesthetised with 2% isoflurane and 0.3 L/min of oxygen. The regions of interest (ROI) image processing and analysis were visualised by Live Image 4.5 software. To quantitative analysis, the ROIs of the tumour were evaluated with a white light image, and muscle regions of similar size (opposite positioning with the tumour) were selected as muscle ROI. The background value is subtracted from each of the luminescence images. The average of the fluorescence signal within each ROI was calculated. After the mice were sacrificed, tumours and organs were fixed with 10% formalin solution.

The *in vivo* toxicity of KL-6 was analysed through H&E histological staining of the major organs. The heart, liver, spleen, lung, kidney, and brain were embedded in tissue freezing medium, frozen to −80 °C and cut in a cryostat, fixed, blocked, and stained in Mayer’s Haematoxylin for 10 min, and further stained with eosin for <1 min. After staining, the slides were dehydrated, mounted, and analysed using an inverted microscope.

#### Western blotting analysis

MKN1 cells were plated in 6-well dishes (3 × 10^5^ cells/well) overnight and then and incubated with compound KL-6 at different concentrations (0, 1, 2, or 5 µM) for 24 h. RIPA buffer containing an inhibitor of serine protease and phosphatase inhibitor was used to lyse the cells. The cell lysates were quantified, separated by an SDS-PAGE gel, and transferred to a PVDF blotting membrane (GE Healthcare, USA), which was further blocked by 5% milk and incubated with primary antibodies, including anti-GAPDH (#5174S), anti-STAT3 (#12640S), and anti-Phospho-Stat3 (Tyr705; # 9145S; Cell Signalling Technology, Shanghai, China) at 4 °C overnight. The membranes were washed with TBST and incubated with a second antibody, antirabbit (#7074) at room temperature for 2 h. The antibody-protein complexes were detected using an ECL luminescence reagent (Biosharp, Shanghai, China). The experiment was independently conducted at least three times.

### Molecular docking and molecular dynamics (MD) simulation procedures

The crystal structure of STAT3 (PDB code: 1BG1) was achieved from the RCSB protein data bank (http://www.rcsb.org/). The modelling experiment described in this study was performed by the docking programs of Autodock vina. Compound KL-6 was drawn by ChemBio3D Ultra 14.0 software, and the energy was minimised utilising this software. According to the reported data^23^, the search grids corresponding to STAT3 binding site were identified as centre_x, 102.429; centre_y, 71.634; and centre_z, 65.747; and dimensions were: size_x, 15; size_y, 15; and size_z, 15. To increase the accuracy of docking, the value of exhaustiveness was set to 20. The default parameters were used if it was not mentioned. The best-fit complex was visually analysed on Chimaera 1.14 and PyMoL 2.4.0 software.

To verify whether the binding model was stable, MD simulation was carried out using the GROMACS package. Proteins were described via ff14SB, and the small molecule was assigned via gaff2. The complex was solvated in a water box with TIP3P model and periodic boundary conditions (PBCs) were applied in all directions. PBC were employed to avoid edge effects in MD simulations. The Na^+^ and Cl^–^ counter ions were added to neutralise the system. The long-range electrostatic interactions were calculated by the particle mesh Ewald method. Energy minimisation was performed to clear poor contacts, followed by 100 ps NVT and 100 ps NPT equilibrations. Finally, a 100 ns MD production simulation was performed with a 2 fs time step at constant temperature (300 K) and pressure (1 atm).

## Results and discussion

### Synthesis of benzochalcone derivatives

**Scheme 1.**
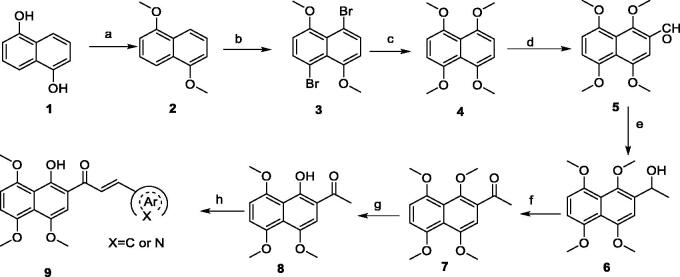
Reagents and conditions: (a) KOH, (CH_3_)_2_SO_4_, rt. for overnight; (b) CH_3_CN, NBS, –10 °C, 12 h; (c) CH_3_ONa, CuI, reflux for overnight; (d) POCl_3_, DMF, reflux for 12 h; (e) CH_3_MgI, Et_2_O, NH_4_Cl, rt for 20 h; (f) MnO_2_, DCM, reflux for 12 h; (g) AlCl_3_, CH_3_CN, 60 °C for 3 h; (h) KOH in EtOH, reflux for 5 h.

Based on our laboratory reported work^9^^,^^10^^,^^13^, we have successfully presented a synthetic route for the preparation of these target compounds. As outlined in Scheme 1, Methylation of commercially available 1,5-dihydroxynaphthalene (**1**) followed by bromination and methoxylation gave 1,4,5,8-tetramethoxynaphthalene (**4**). Vilsmeier reaction of the intermediate **4** with *N*,*N*-dimethylformamide (DMF) and phosphorus oxychloride (POCl_3_) provided 2-formyl-1,4,5,8-tetramethoxynaphthalene (**5**), followed by reacting with Grignard reagent (CH_3_MgI, prepared from CH_3_I and Mg in diethyl ether) to give the 1–(1,4,5,8-tetramethoxynaphthalen-2-yl)ethan-1-ol (**6**). Oxidation of the intermediate **6** by active manganese dioxide (MnO_2_) afforded the corresponding 1–(1,4,5,8-tetramethoxynaphthalen-2-yl)ethan-1-one (7), which was subjected to selective demethylation in the presence of aluminium chloride (AlCl_3_) to yield 1–(1-hydroxy-4,5,8-trimethoxynaphthalen-2-yl)ethan-1-one (**8**). Finally, the target compounds were obtained by Claisen-Schmidt condensation of the key intermediate **8** and different aromatic (heterocyclic) aldehyde in the presence of ethanolic KOH.

### Antiproliferation activity of compounds 9a–9t against gastric cancer cells

Immortal proliferation is a significant feature of cancer cells; thus, we initially evaluated the antiproliferative activity of all the synthesised compounds against five gastric carcinoma cell lines (HGC27, MKN28, AZ521, AGS, and MKN1). The Cell Counting Kit-8 assay (CCK-8) was used to determine their IC_50_ values through incubating all the tested compounds with the gastric carcinoma cells at various concentrations and specific time. Cisplatin (DDP), a widely used gastric cancer chemotherapy^24^, was used as a positive control. In light of these gastric carcinoma cell lines overexpressing STAT3, C188-9 (known as TTI-101, a STAT3 inhibitor in clinical trial) was also served as a positive drug in this study.

As shown in Table 1, these compounds exhibited varying degrees of cytotoxic effect on the selected tumour cells, and most of them were more potent than cisplatin and C188-9. Compound **9a** bearing a phenyl ring showed high inhibitory activity against all the tested cell lines except MKN28 with IC_50_ values below 10 μM. However, introduction of one more phenyl ring into **9a** provided compounds **9b–9d**, which caused a differential decrease in antiproliferative activity towards the tested five cell lines. Especially, the biphenyl ring derivative displayed a dramatic drop of cytotoxic activity with IC_50_ values over 50 μM for all the tested cells. From these results, we speculate that the introduction of phenyl ring would lead to increased hydrophobicity, which may be correlated with their decreased efficiency in cytotoxicity.

**Table 1.** The structures of compounds **9a**–**9t** and their antiproliferation activity towards five human gastric cell lines (HGC27, MKN28, AZ521, AGS, and MKN1). 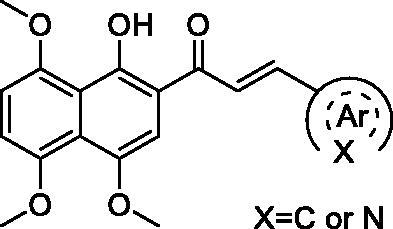

Compd.	Ar	IC_50_ (μM)				
		HGC27	MKN28	AZ521	AGS	MKN1
**9a**	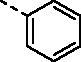	7.49	27.99	5.89	9.01	7.22
**9b**	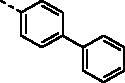	>50	>50	>50	>50	>50
**9c**	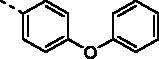	15.33	19.21	>50	>50	22.03
**9d**	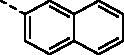	14.29	49.36	13.27	9.46	10.10
**9e**	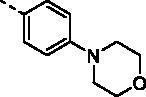	12.66	>50	11.22	12.72	49.29
**9f**	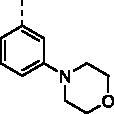	2.63	6.31	32.04	38.11	2.70
**9g**	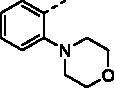	13.82	41.48	9.23	8.60	19.91
**9h**	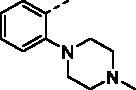	1.34	4.28	10.62	11.01	17.09
**9i**	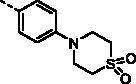	9.68	18.24	48.79	48.80	24.6
**9j**	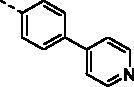	11.43	26.07	8.35	3.69	9.26
**9k**	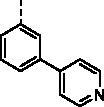	1.63	9.75	2.98	2.14	2.82
**9l**	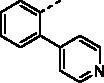	9.55	12.02	4.95	6.68	8.30
**9m**	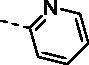	1.18	13.49	10.03	1.39	6.20
**9n**	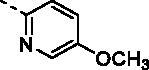	6.35	10.26	13.17	12.44	4.03
**9o (KL-6)**	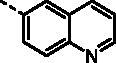	0.63	6.43	1.14	3.25	1.96
**9p**	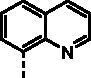	1.59	5.31	9.55	9.63	1.91
**9q**	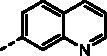	1.43	3.08	12.44	9.83	1.53
**9r**	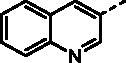	4.38	2.75	13.55	11.89	6.30
**9s**	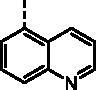	1.33	3.36	10.99	9.62	1.67
**9t**	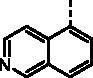	5.83	10.31	25.85	15.21	8.79
DDP	–	>50	>50	6.08	42.13	28.83
C188-9	–	21.06	40.01	4.56	9.99	10.21

With the great advantages of heterocycles in mind, introducing heterocycles into compound **9a** is proposed to improve their physical properties, and antiproliferative activity. We first introduced the aliphatic heterocycle into the different positions of phenyl ring. The antitumour activity of compounds **9e–9g** was generally improved to a certain extent, when a morpholine group was incorporated into the *ortho*-, *meta*-, and *para*-position of the phenyl ring of **9a**. Particularly, the *meta*-morpholine substituted derivative **9f** performed remarkable antitumour activity against HGC27, MKN28, and MKN1 with IC_50_ values of 2.63, 6.31, and 2.70 μM, respectively. Notably, replacement of the “O” atom of morpholine moiety in **9g** with a “*N*-methyl” gave compound **9h**, which showed 10-fold more potent cytotoxicity towards HGC27 and MKN28 than **9g**, with IC_50_ values of 1.34 and 4.28 μM, respectively. However, the antitumour activity was not obviously improved or even reduced in different degrees when the “O” atom of morpholine moiety in **9e** was replaced by a sulphone group (**9i**). These results highlight the importance of nitrogen-containing heterocycle and its position in maintaining the anticancer activity.

Based on the above hypothesis, we then introduced a nitrogen-containing aromatic heterocycle (pyridine ring) into the *ortho*-, *meta*-, and *para*-position of the phenyl ring of **9a**. Consistently, among compounds **9j–9l**, the *meta*-pyridyl substituted derivative **9k** also had the most favourable activity against different gastric carcinoma cells (except MKN28 cell line, IC_50_ values ranging from 1.63 to 2.98 μM), which was more potent than the corresponding morpholine substituted derivative **9f**. The structural features may suggest that the substituent at *meta*-position of phenyl ring is favourable for cytotoxicity towards gastric cancer cells. Removal of the phenyl ring in compounds **9j–9l** produced the pyridyl substituted derivative **9m**, which showed a slight increase in blocking the growth of HGC27 and AGS, with IC_50_ values around 1 μM. However, addition of a methoxy group to the pyridyl ring led to more than six-fold loss in potency against HGC27 and AGS cell lines (IC_50_ values of 1.63 and 2.98 μM, respectively). Further modification of compounds **9j–9l** was carried out by fusion of their pyridyl rings into the phenyl ring, and the obtained quinoline derivative **9o** (KL-6) generally showed strong antiproliferation activity against all the tested cells (HGC27, MKN28, AZ521, AGS, and MKN1) with IC_50_ values of 0.63, 6.43, 1.14, 3.25, and 1.96 μM, respectively. With the above result in hand, compounds **9p–9t** were synthesised to assess the effect of the different position substituted quinoline on the cytotoxicity. Unfortunately, the cytotoxicities of quinoline derivative **9p–9s** were not improved (IC_50_ values ranging from 1.33 to 13.55 μM towards the selected cells). Even worse, isoquinoline derivative **9t** suffered a somewhat loss of antiproliferation activity. Above all, the SAR study highlighted the importance of aromatic heterocycle in antiproliferative activity, and the quinolin-6-yl substituted derivative KL-6 was identified as the most potent one in this series. Given that KL-6 also considerably inhibited the growth of MKN1 cells, and we have successfully established its orthotopic tumour model, we performed further biological evaluation of compound KL-6 based on MKN1 cells.

### KL-6 inhibits colony formation and induces apoptosis of MKN1 cells

**Figure 2.**
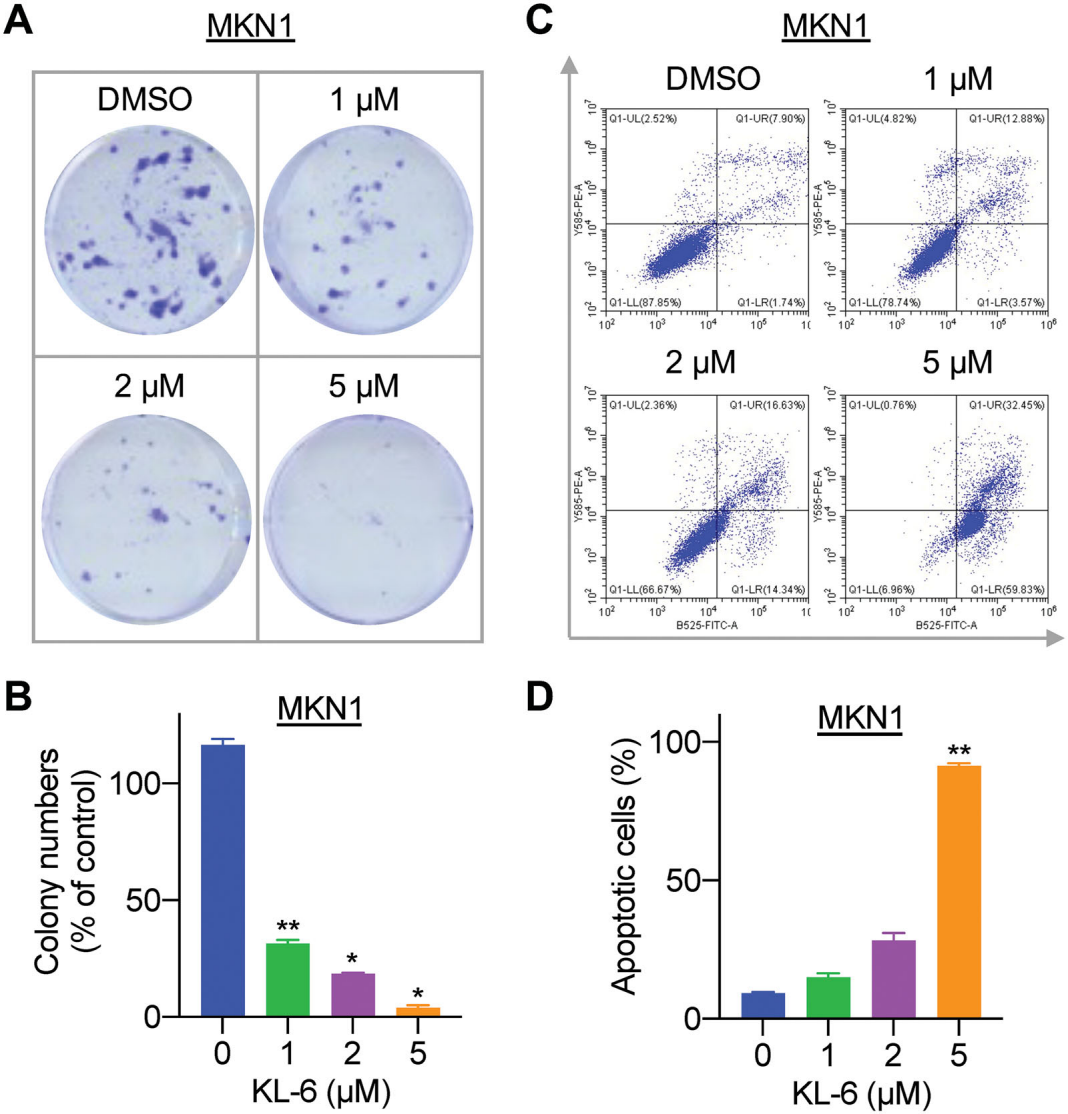
Effects of KL-6 on gastric cancer cell proliferation and apoptosis. (A) Effects of compound KL-6 on clonogenicity of MKN1 at indicated concentrations. (B) Number of the colony MKN1 cells (*: *p* < 0.05, **: *p* < 0.01). (C) Flow cytometric analysis of MKN1 cells treated with tested compound with indicated concentrations for 48 h. (D) Percentage of the apoptotic cells (**: *p* < 0.01).

Colony formation assay is used to assess the ability of cells to grow into a colony and to undergo “unlimited” divisions, which is an important indicator of a cancer cell to attach, survive, and proliferate^25^^,^^26^. To investigate the inhibitory effect of the most potent molecule KL-6 on the clonogenicity of MKN1 cells, a plate colony formation assay was performed. As shown in Figure 2(A,B), the colony number and size significantly decreased in a concentration-dependent manner after exposure of MKN1 cells to KL-6 at the concentration over 1 μM. The results demonstrated that KL-6 could effectively inhibit the proliferation of gastric cancer.

Initiation of apoptotic signals pathway and induction of apoptosis have been considered as an effective approach for tumour treatment^27^. Owing to the potent inhibitory activity against MKN1 cells, compound KL-6 was selected to investigate its ability to induce cancer cells apoptosis by Annexin V-FITC/PI staining. As shown in Figure 2(C,D), flow cytometric analysis results indicated that after various concentrations (1, 2, and 5 μM) of compound KL-6 treatment for 48 h, the total percentage of early and late apoptosis of MKN1 cells increased to 16.45%, 30.97%, and 92.28%, respectively, with the increasing concentration of compound KL-6, especially at the concentration of 5 μM. Accordingly, these results suggested that compound KL-6 could note perform the apoptosis-inducing activity in a concentration-dependent manner in MKN1cells.

### KL-6 inhibits migration and invasion of MKN1 cells

**Figure 3.**
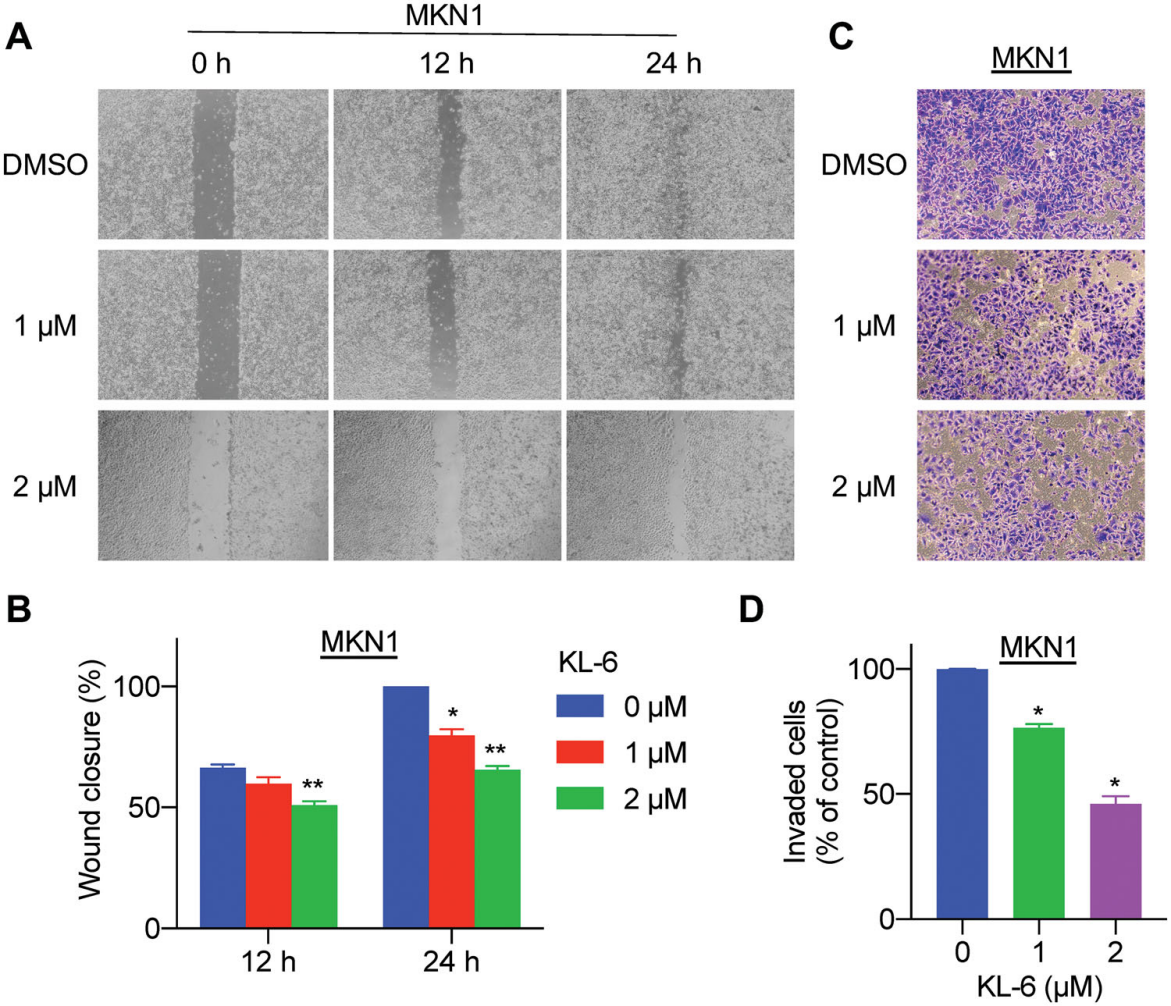
Compound KL-6 interfered with the migration and invasion of gastric cancer cells. (A) Representative photomicrographs of MKN1 cells treated with compound KL-6 at indicated concentrations for 12 h or 24 h by wound healing assay. (B) The percentage of the wound closed area (*: *p* < 0.05, **: *p* < 0.01). (C) Transwell assay of MKN1 cells after treatment for 48 h with compound KL-6 at indicated concentrations. (D) Percentage of the invaded cells in the transwell assay (*: *p* < 0.05).

Cells migration and invasion are important biological characteristics of tumour metastasis, which contributes to the malignant and recurrence problems in cancer therapy^28^. To further investigate whether compound KL-6 could subdue this transition process, we evaluated the migration ability of MKN1 cells by wound healing and transwell assays. The wound healing assay results showed that the scratched area of untreated cells decreased notably compared with the treated group after 24 h (Figure 3(A,B)), suggesting that the migration of cells was decreased significantly and concentration-dependently in the KL-6 treated group. Similarly, compound KL-6 also exhibited strong antimigratory potency in the transwell migration models. As seen in Figure 3(C,D), a conspicuous and concentration-dependent reduction of invaded cells can be observed after treatment of compound KL-6. In general, KL-6 had an outstanding inhibitory potency on the migration and invasion of the tested MKN1 human gastric cancer cells.

### *In vivo* antitumour activity of KL-6 on MKN1 orthotopic tumour model

**Figure 4.**
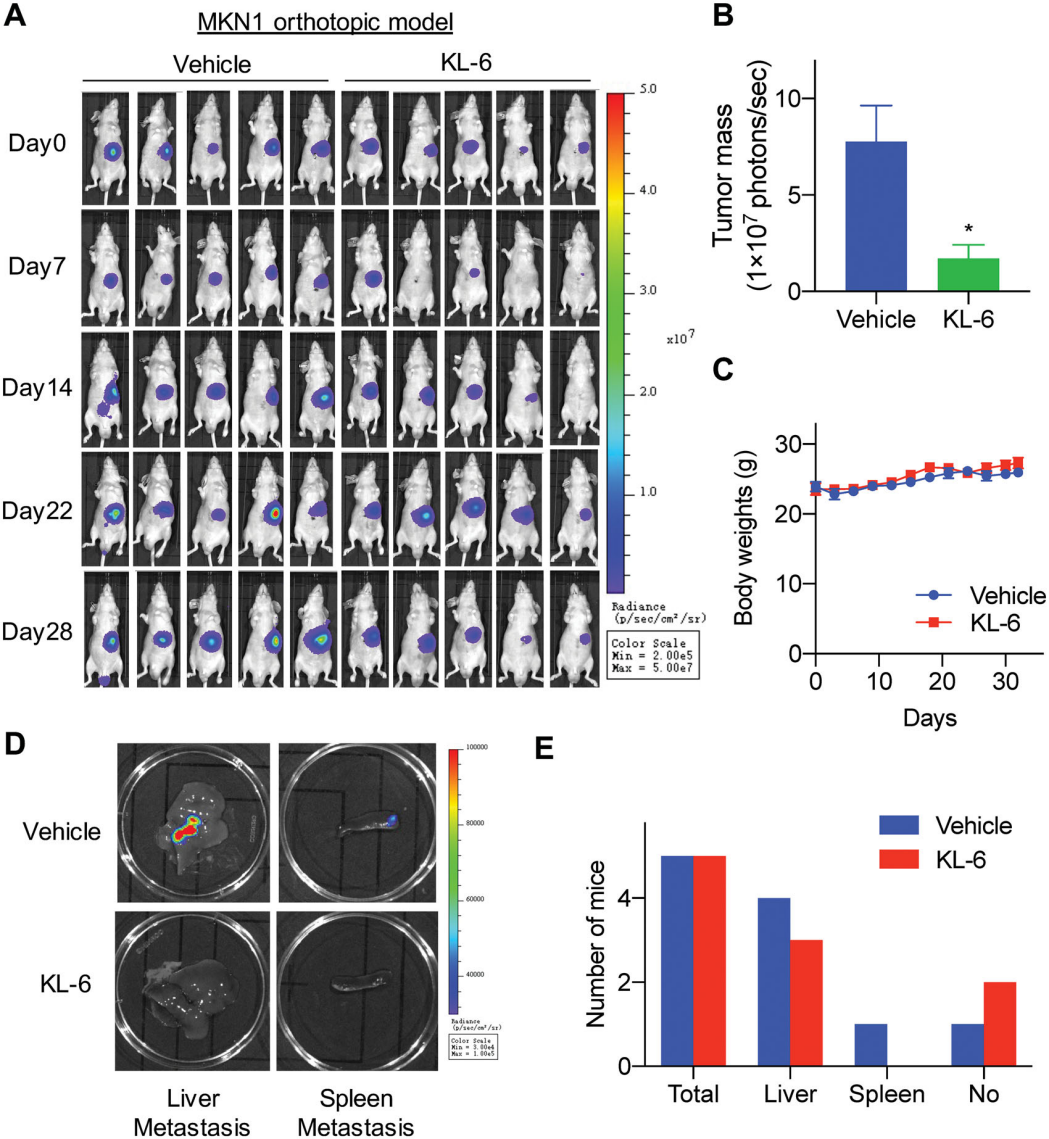
*In vivo* antitumour effect of compound KL-6 in an orthotopic xenograft model of MKN1. (A) Bioluminescence signal of experimental groups observed at indicated days. (B) Tumour growth (expressed as Luciferase Average radiance) measurements at the termination of the experiments, *: *p* < 0.05. (C) Body weight throughout the experimental period. (D) Tissues (liver and spleen) were imaged to detect metastatic lesions. (E) The numbers of mice with metastasis to the liver, and spleen.

Inspired by the potent anticancer activity of compound KL-6 against gastric cancer cells, we further evaluated its antitumour effect *in vivo* on an orthotopically xenografted BALA/c nude mice model bearing MKN1 tumour. As shown in Figure 4(A,B), the tumour growth of mice in the treated group was remarkably inhibited, with the tumour growth inhibition (TGI) rate of 78% at an intraperitoneal dose as low as 5 mg/kg once daily for 28 days. Importantly, no significant loss in the average body weight was observed compared with the vehicle group, implying no noticeable adverse events of compound KL-6 in mice (Figure 4(C)).

The effect of KL-6 on metastasis in mice bearing MKN1 orthotopic tumours was also investigated. As disclosed in Figure 4(D,E), the data collected at necropsy revealed that four of five vehicle-treated mice developed metastatic lesions in the liver, whereas the incidence of liver metastasis in KL-6-treated mice was decreased to 3/5. Furthermore, none of the mice had metastatic lesions in the spleen in the treatment group. Notably, only one of five vehicle-treated mice showed no metastatic lesions in the liver or spleen, while the incidence of no metastasis in KL-6-treated mice was increased to 2/5. To a certain extent, the results suggested that compound KL-6 has the potential to inhibit metastasis in gastric cancer.

**Figure 5.**
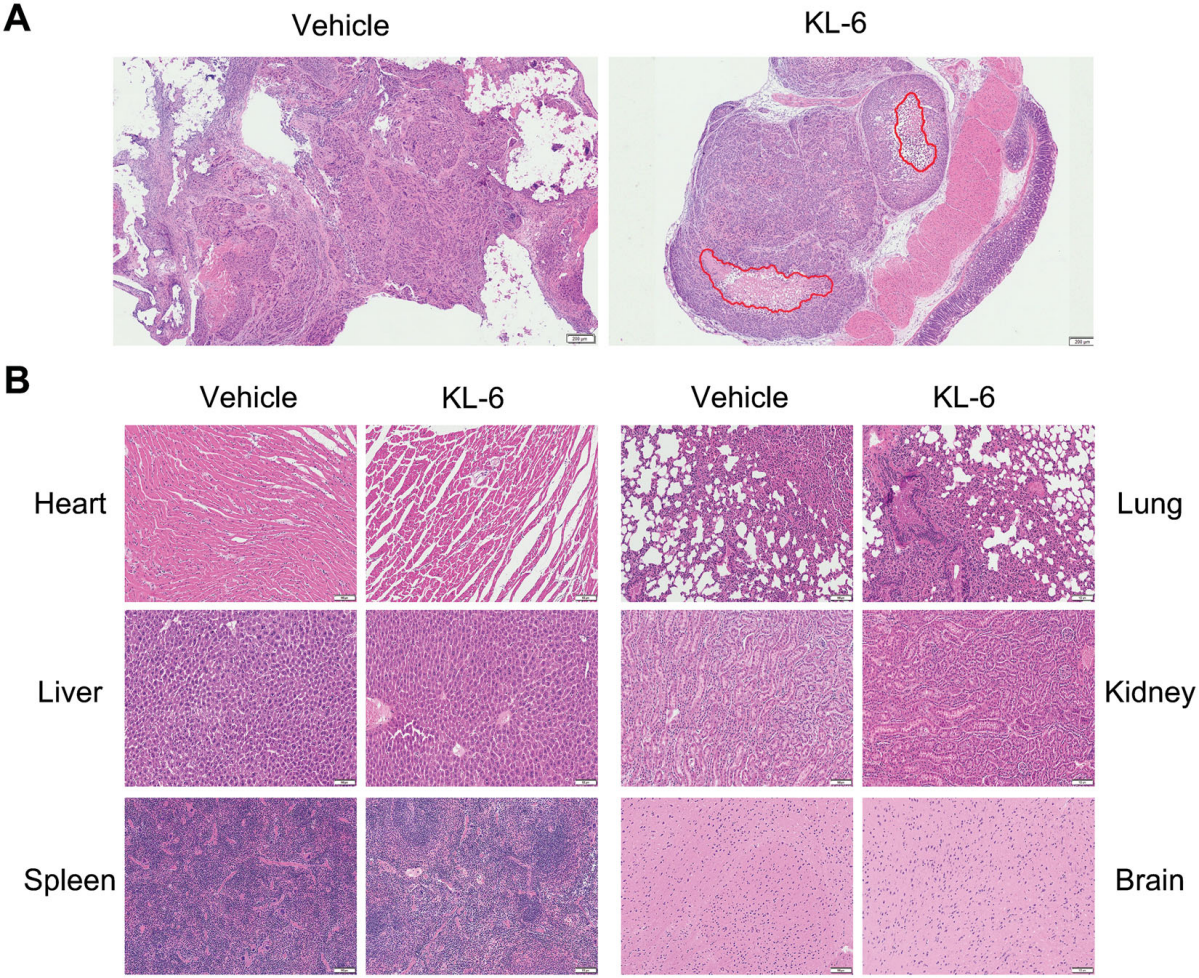
H&E staining of tumour and major organs sections. (A) Tumour stained with H&E, and red circle represents necrosis (Scale bar = 200 μm). (B) Histological section of anatomical heart, liver, spleen, lung, kidney, and brain (Scale bar = 100 μm).

Of note, KL-6 can induce necrosis in the tumour tissue as evidenced by H&E staining (Figure 5(A)), whereas no necrosis can be detected in the control group, highlighting the anticancer activity of KL-6. In addition, the pathological change in major organs was examined by H&E staining to further confirm the toxicity profile of KL-6. As presented in Figure 5(B), similar to the control group, the histology of the heart, liver, spleen, lung, kidney, and brain indicated that the cells of organ tissues from the KL-6 treated group maintained a complete morphology, without obvious cellular inflammatory, oedema, or necrosis, suggesting no obvious toxicity of KL-6 to the major organs.

### KL-6 shows anticancer activity on gastric cancer cells partly through blocking STAT3 tyrosine phosphorylation

STAT3 is a transcription factor that regulates various biological processes^29^. Inactive STAT3 is localised in the cytoplasm. Once stimulated by growth factors and cytokines, as well as oncogenic proteins, STAT3 monomer can be phosphorylated at tyrosine residue (Y705). Phosphorylated STAT3 (p-STAT3) proteins can form dimers, which then translocate into the nucleus and bind to DNA, causing cellular antiapoptosis, proliferation, metastasis, and tumour invasion^17^. In normal cells, STAT3 is tightly regulated to maintain a transiently active state, while persistent STAT3 activation occurs frequently in ∼70% of human solid and haematological malignancies, associating with a poor prognosis and tumour progression^17^. Therefore, it has spurred considerable efforts over the last two decades to clinically exploit the beneficial effects of blocking STAT3 in human malignancies.

**Figure 6.**
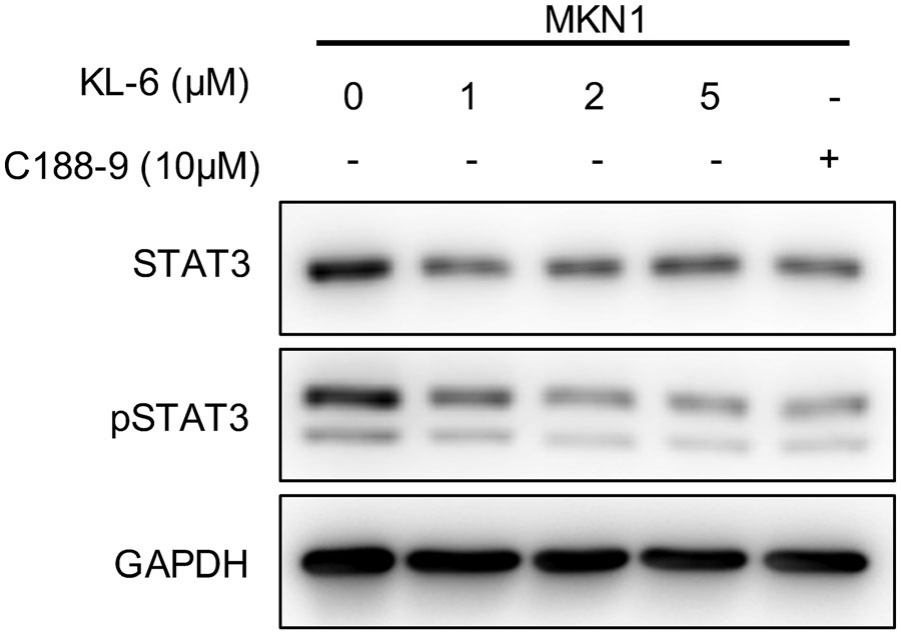
Western blot analysis of KL-6 mediated effects on STAT3 and p-STAT3 inhibition in MKN1 tumour cells.

Based on the observations that: (1) STAT3 is aberrantly active in gastric cancer cell lines; (2) KL-6 showed potent anticancer activities towards gastric cancer cell lines involving antiproliferation, antimetastasis, apoptosis-inducing, and suppression of colony-forming; (3) STAT3 has been reported as one of the pharmacological targets of chalcone scaffold, we therefore explored whether the anticancer properties of KL-6 may be related to the blockade of STAT3 signalling pathway. A western blot assay was performed to investigate the expression level of total STAT3 and phosphorylated STAT3 (p-STAT3) after incubation of various concentrations (1, 2, and 5 μM) of compound KL-6 with MKN1 cells for 24 h. As expected, KL-6 markedly suppressed STAT3 phosphorylation (p-STAT3^Y705^) in a concentration-dependent manner without significant effect on total STAT3 protein level (Figure 6). Furthermore, KL-6 was much more potent than the positive STAT3 inhibitor C188-9 in inhibiting the phosphorylated STAT3 (The level of inhibition mediated by 2 μM KL-6 is similar to that produced by 10 μM C188-9). These data demonstrated that the anticancer effect caused by compound KL-6 in gastric cancer cells was partly mediated by the STAT3 signalling pathway.

### Molecular docking prediction of KL-6 with STAT3 and MDs simulation

**Figure 7.**
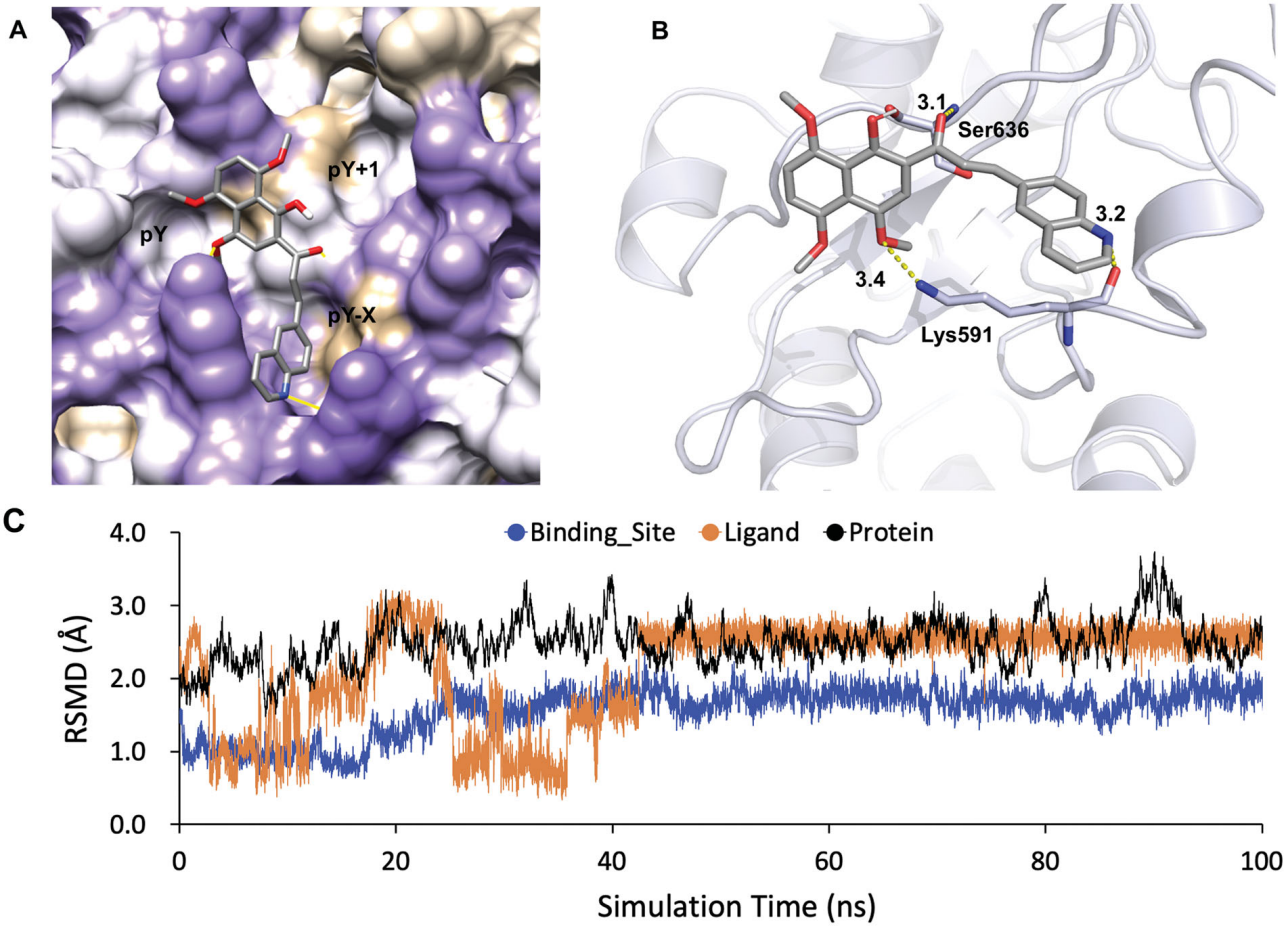
Docking model of KL-6 binding to the STAT3 SH2 domain (PDB: 1BG1) generated by Autodock Vina. (A) KL-6 was coloured by atom type. The surface of SH2 domain was coloured according to hydrophobic property (the most polar residues are medium purple while the most hydrophobic are tan). (B) STAT3 protein was represented as cartoon. The key residues were shown as sticks. Hydrogen bonds were represented by yellow dashes. (C)The RMSDs of the STAT3- KL-6 complex obtained during 100 ns of MD simulation.

To gain insight into the plausible binding modes and rationalise the observed efficiency of STAT3 inhibition by the compound KL-6, a molecular docking study was performed from the Protein Data Bank using crystal structure data for STAT3 (PDB entry 1BG1)^30^. STAT3 contains several structural motifs such as the N-terminal domain, the coiled-coil domain, the DNA-binding domain, the linker domain, the Src homology 2 (SH2) domain, and the transactivation domain. The homodimerisation of STAT3 is mediated by a reciprocal pY705-SH2 domain interaction. In the STAT3 SH2 domain, three adjacent binding subpockets were explored for drug targeting, including (1) the phosphorylated Tyr705 (p-Tyr705)-binding pocket (also named pY subpocket, residues 591, 609–620); (2) the Leu706 subsite (also named pY + 1 subpocket, residues 626–639); and (3) a side pocket (also named pY-X subpocket, residues 592–605). Among them, pY site is the most important for inhibitor binding to disrupt STAT3 phosphorylation and dimerisation. As illustrated in Figure 7, the *in silico* docking calculations suggested that KL-6 was well fitted into the SH2 domain of STAT3 with the naphthalene fragment binding to the key pY pocket and the quinoline moiety occupying the pY-X pocket (Figure 7(A)). Structurally, the methoxyl, carbonyl, and quinolinyl groups formed three hydrogen bonds with residues Lys591 and Ser636 with distances of 3.4, 3.1, and 3.2 Å, respectively (Figure 7(B)). Herein, it may also provide a partial explanation for the importance of the 6-quinolinyl group in KL-6 for anticancer activity. Additionally, extra hydrophobic contacts caused by the methyl group and naphthalene fragment were another crucial factor to the STAT3 inhibitory effect of KL-6.

To further explore whether the ligand binding to STAT3 was stable, 100 ns MD simulation has been performed and the root mean square deviations (RMSD) of ligand (in orange colour), binding site (in blue colour, the residue around ligand < 4.50 Å) and protein (in black colour) against simulation time were displayed in Figure 7(C). It showed that the ligand was accommodated in a stable conformation and reached the equilibrated state with the RMSD value of 2.50 Å after ∼45 ns simulations. Meanwhile, the RMSD value of binding site was less than that of ligand. Possibly, the ligand could adopt the suitable binding mode in the binding site of STAT3 predicted via molecular docking and stabilise the conformation of the binding site. The RMSD values of protein were larger than that of ligand and binding site, which may be due to the flexibility of STAT3 loop regions. In general, MD simulation indicated that the ligand could bind to the binding pocket in STAT3 via the suitable and stable conformation.

### Drug-like properties of KL-6

In view of the promising anticancer activity of KL-6 against gastric cancer cells, the druglike properties of KL-6 were calculated using Molinspiration tool (https://www.molinspiration.com). Interestingly, the parameters of compound KL-6 including the molecular weight (MW = 415.44), hydrogen bond donors (nOHNH = 1), hydrogen bond acceptors (nON = 6), rotatable bonds (nrotb = 6), and molinspiration predicted log *P* (mi log *P* = 4.69) were consistent with Lipinski’s rule of five, while the mi log *P* of C188-9 (mi log *P* = 5.03) was slightly deviated from the acceptable range (Table 2). In addition, the topological polar surface area (tPSA) value of KL-6 was predicted as 77.89 Å^2^, which was slightly better than that of C188-9, suggesting the potential advantage for intestinal absorption (<140 Å^2^) and inability to pass through the blood-brain barrier (>60 Å^2^). Overall, these calculated parameters showed that KL-6 may possess good drug-like properties.

**Table 2.** Physicochemical parameters of compound KL-6.

Parameter items	KL-6	C188-9
MW (<500 Da)	415.44	471.53
nON (<10)	6	6
nOHNH (<5)	1	3
nrotb (<10)	6	5
mi log P (<5)	4.69	6.27
tPSA (<140 Å^2^)	77.89	95.86

## Conclusion

In summary, a series of benzochalcone analogues involving heterocyclic molecules were synthesised and biologically evaluated for their antitumour activity against gastric cancer *in vitro* and *in vivo*. Most of the compounds exhibited strong antiproliferation activity against the tested gastric carcinoma cell lines (HGC27, MKN28, AZ521, AGS, and MKN1), which were more potent than the positive controls cisplatin and C188-9. The SAR study highlighted the importance of aromatic heterocycle in anticancer efficacy. Among them, the quinolin-6-yl substituted derivative KL-6 inhibited the growth of these five gastric cancer cells with a submicromolar to micromolar range of IC_50_, being the most potent one in this series. The in-depth investigation demonstrated that compound KL-6 could significantly inhibit the colony formation, migration and invasion, and effectively induce apoptosis of MKN1 cells in a concentration-dependent manner. Additionally, STAT3 has been demonstrated to be prominently expressed in various cancer cells, including gastric cancer. Further mechanistic study revealed that KL-6 could concentration-dependently suppressed STAT3 phosphorylation, which may partly contribute to its anticancer activity. The molecular modelling experiment revealed that hydrogen bond was a predominant factor for KL-6 tightly binding to STAT3. Furthermore, the *in vivo* antitumour study on MKN1 orthotopic tumour model showed that KL-6 effectively inhibited tumour growth and metastasis without obvious toxicity. Taken together, this study provided a plausible anticancer mechanism of benzochalcone analogues as compared with our previous work and confirmed that KL-6 could serve as a new starting point for the development of drug candidates for gastric cancer therapy. Attracted by the promising anticancer properties of KL-6, further studies focussing on the deeper pharmacological mechanism and more animal models are now underway in our laboratory.

## Supplementary Material

Supplemental Material
