# Supplementary material for: Discovery of benzochalcone derivative as a potential antigastric cancer agent targeting signal transducer and activator of transcription 3 (STAT3)
Source: J Enzyme Inhib Med Chem. 2022 Jul 18;37(1):2004–16. doi: 10.1080/14756366.2022.2100366 (PMC9297716; doi:10.1080/14756366.2022.2100366)

Supplementary Material

Table of Contents

^{1}H-NMR Spectra, ^{13}C-NMR Spectra and HRMS Spectra of Title Compounds

(*E*)-1-(1-hydroxy-4,5,8-trimethoxynaphthalen-2-yl)-3-phenylprop-2-en-1-one (**9a**)

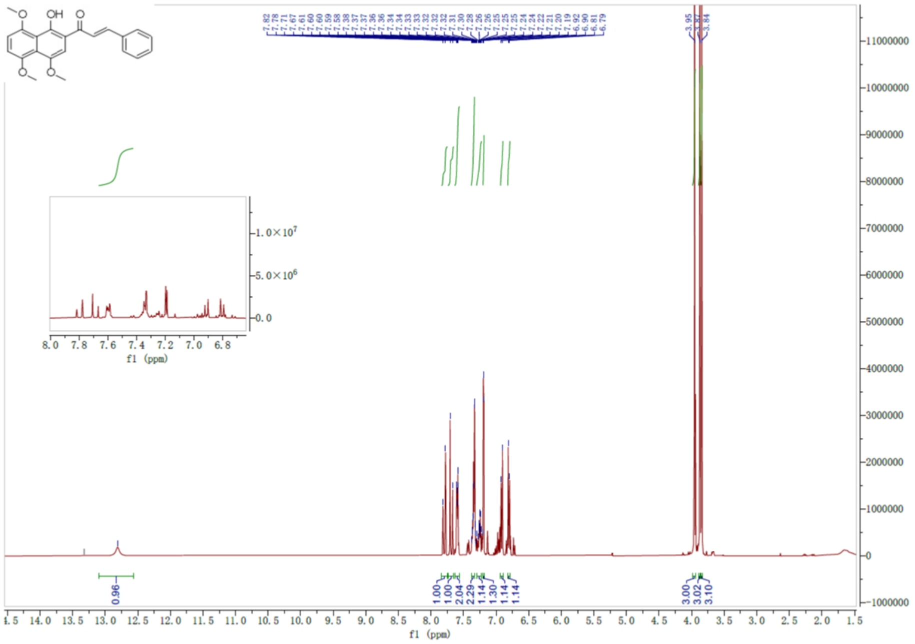

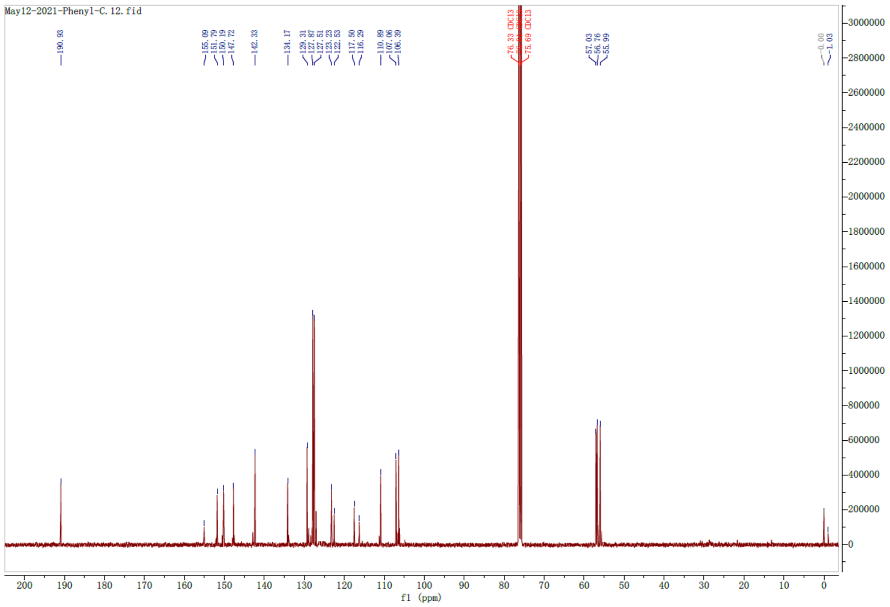

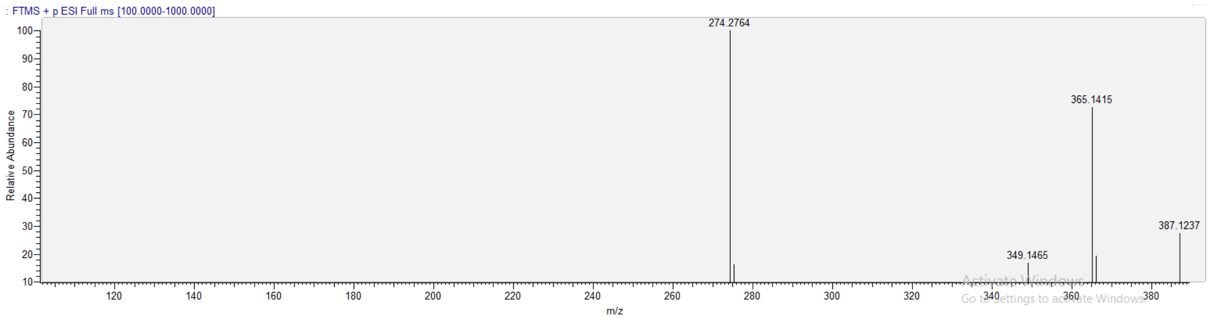

(*E*)-3-([1,1'-biphenyl]-4-yl)-1-(1-hydroxy-4,5,8-trimethoxynaphthalen-2-yl)prop-2-en-1-one (**9b**)

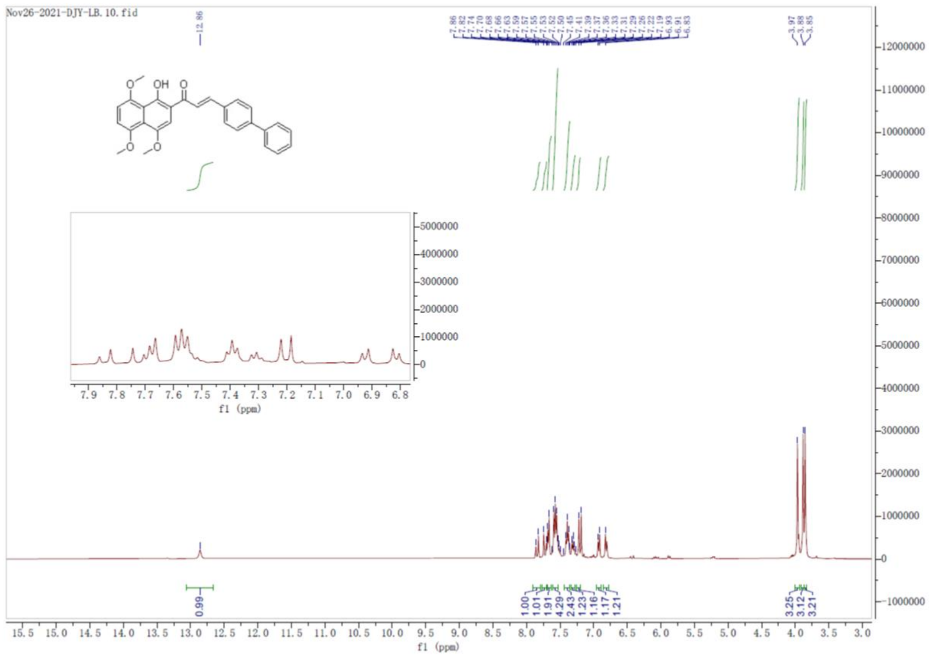

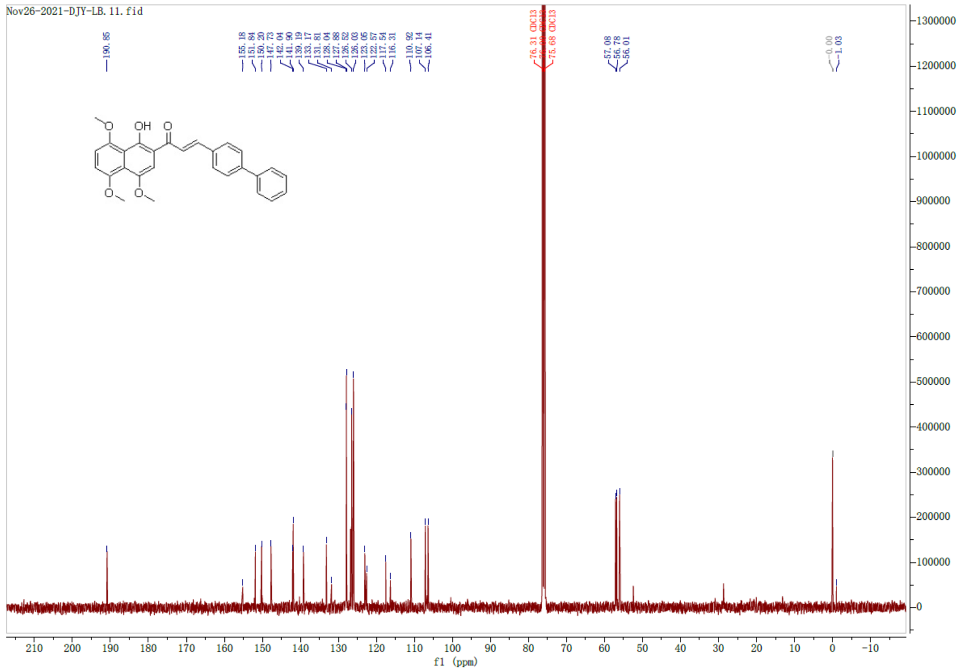

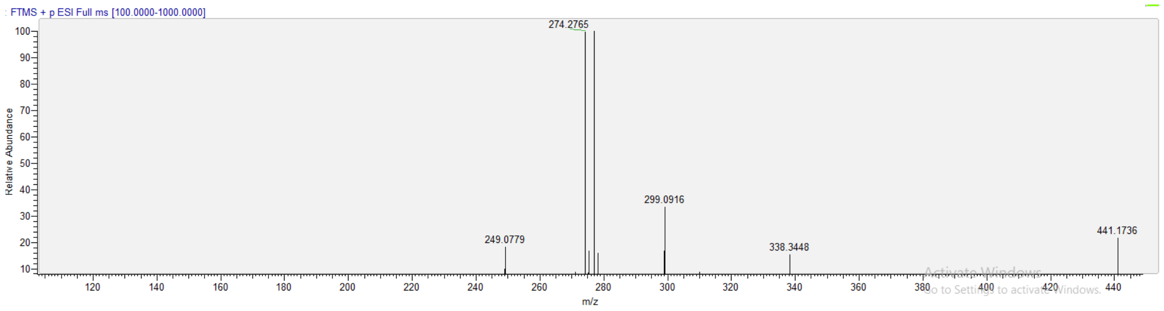

(*E*)-1-(1-hydroxy-4,5,8-trimethoxynaphthalen-2-yl)-3-(4-phenoxyphenyl)prop-2-en-1-one (**9c**)

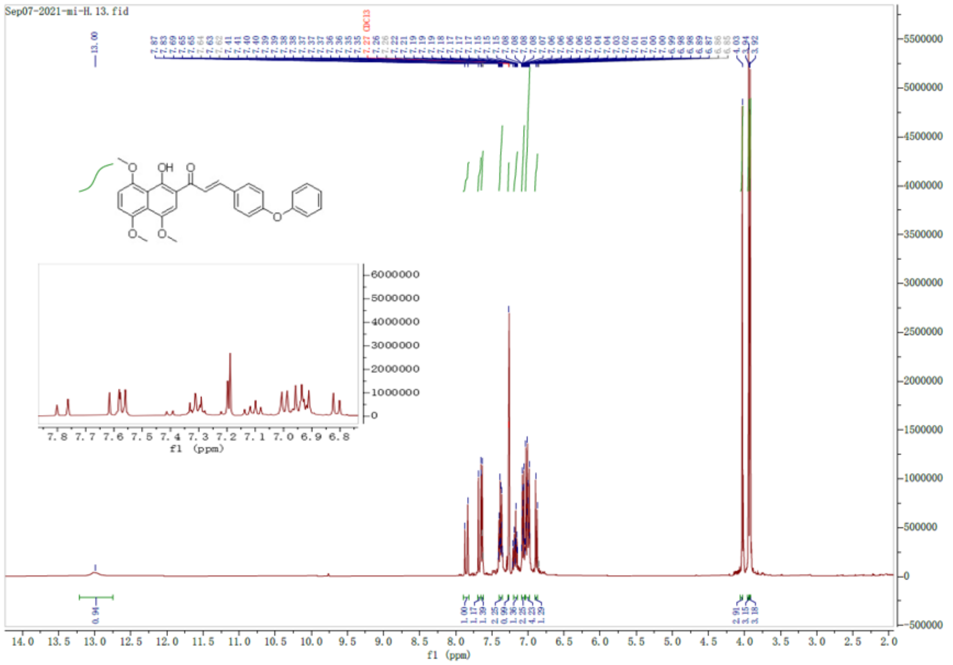

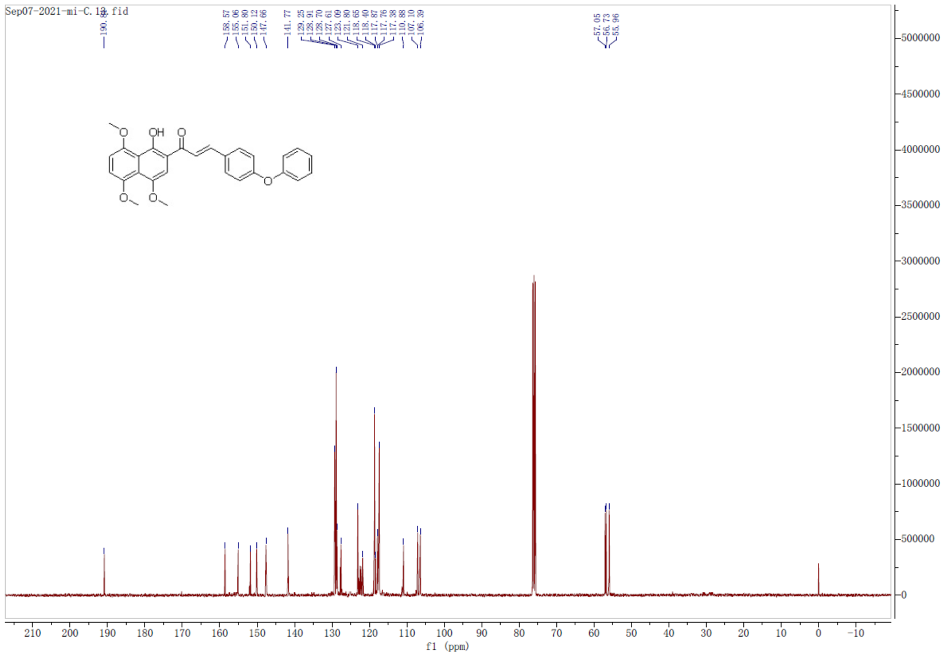

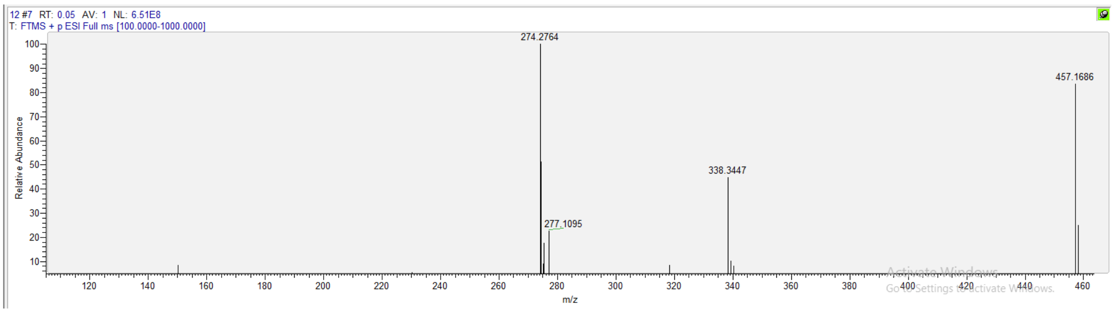

(*E*)-1-(1-hydroxy-4,5,8-trimethoxynaphthalen-2-yl)-3-(naphthalen-2-yl)prop-2-en-1-one (**9d**)

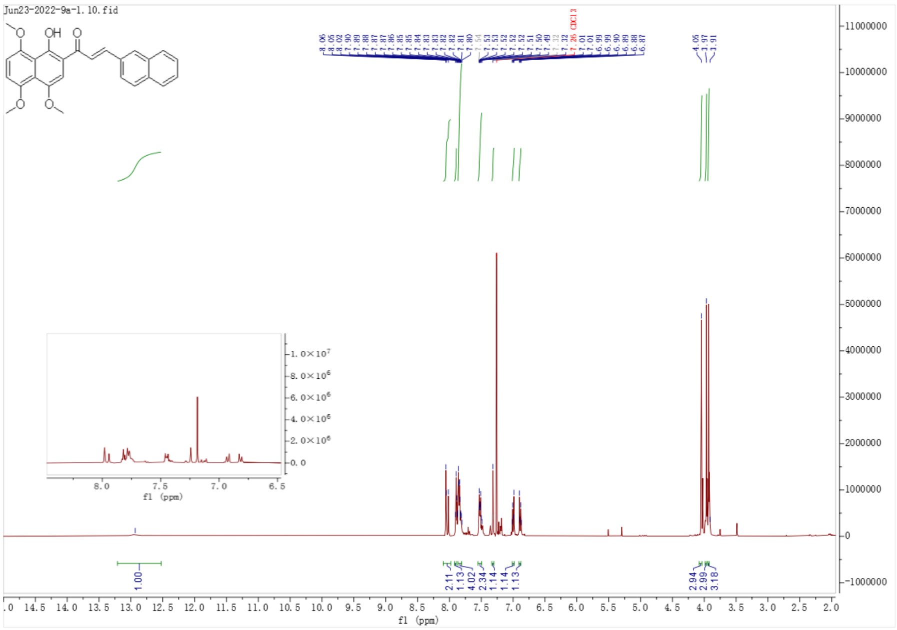

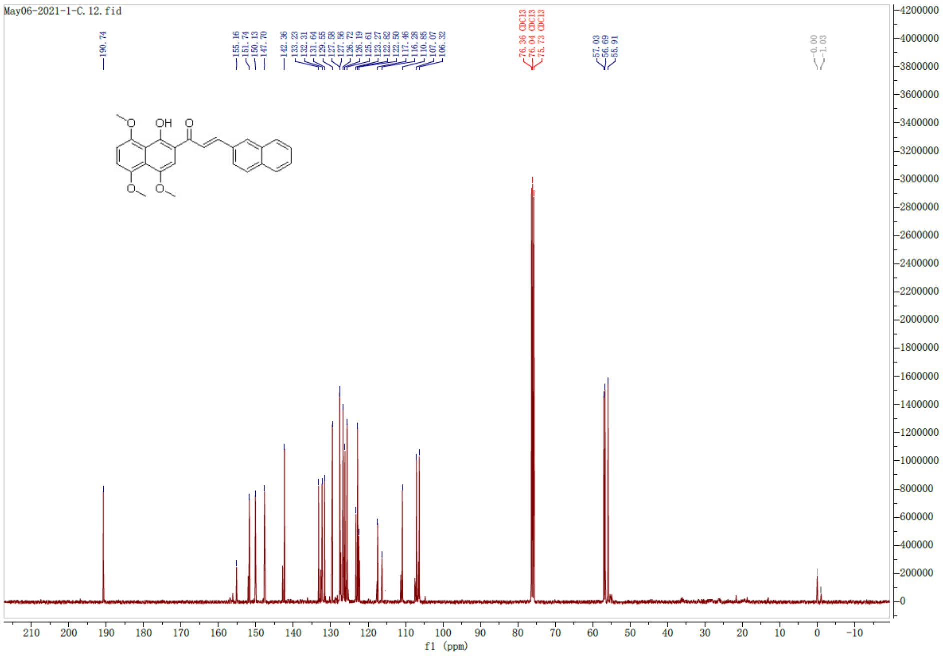

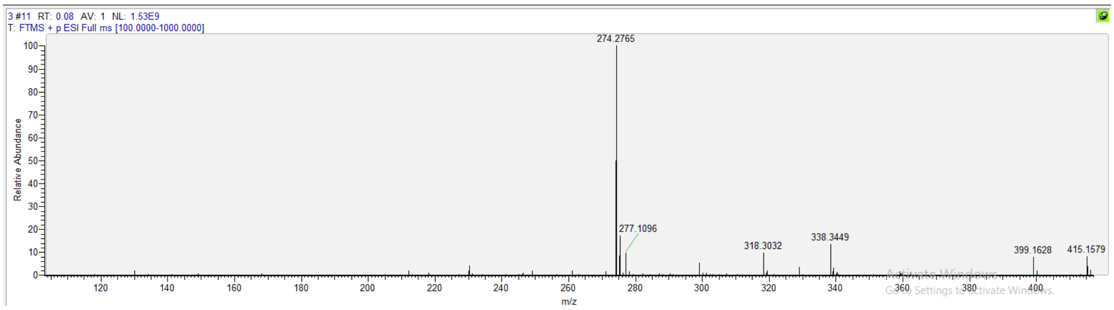

(*E*)-1-(1-hydroxy-4,5,8-trimethoxynaphthalen-2-yl)-3-(4-morpholinophenyl)prop-2-en-1-one (**9e**)

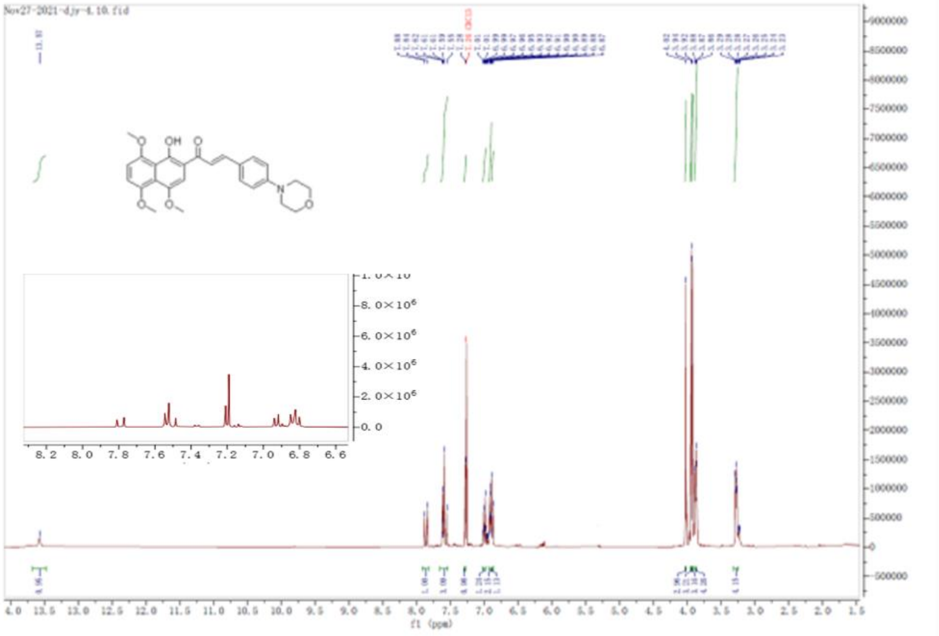

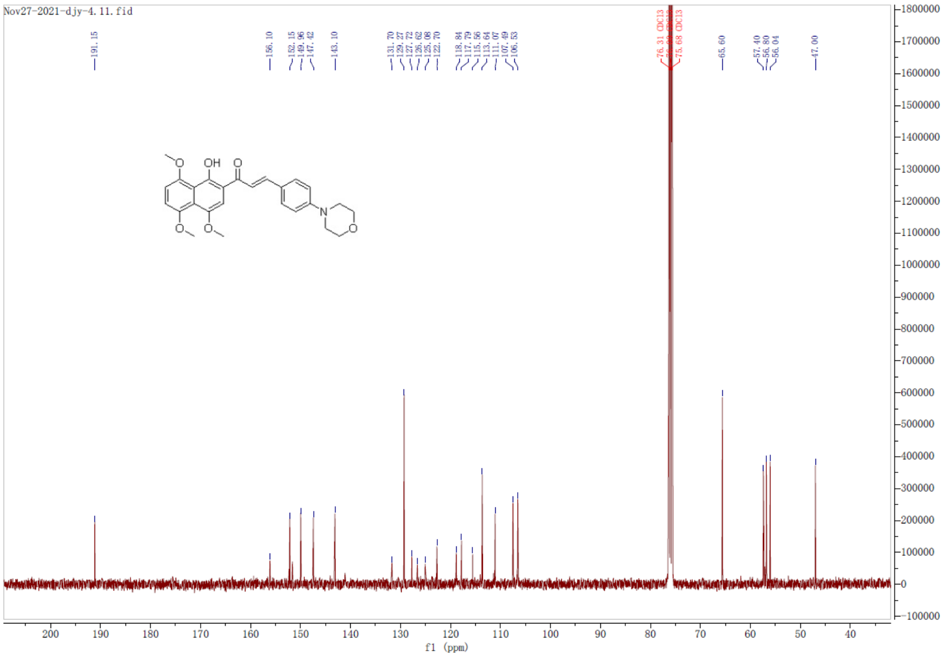

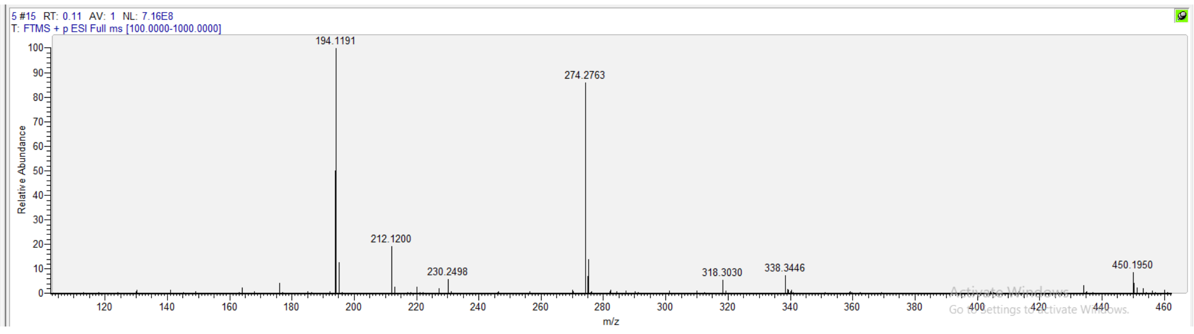

(*E*)-1-(1-hydroxy-4,5,8-trimethoxynaphthalen-2-yl)-3-(3-morpholinophenyl)prop-2-en-1-one (**9f**)

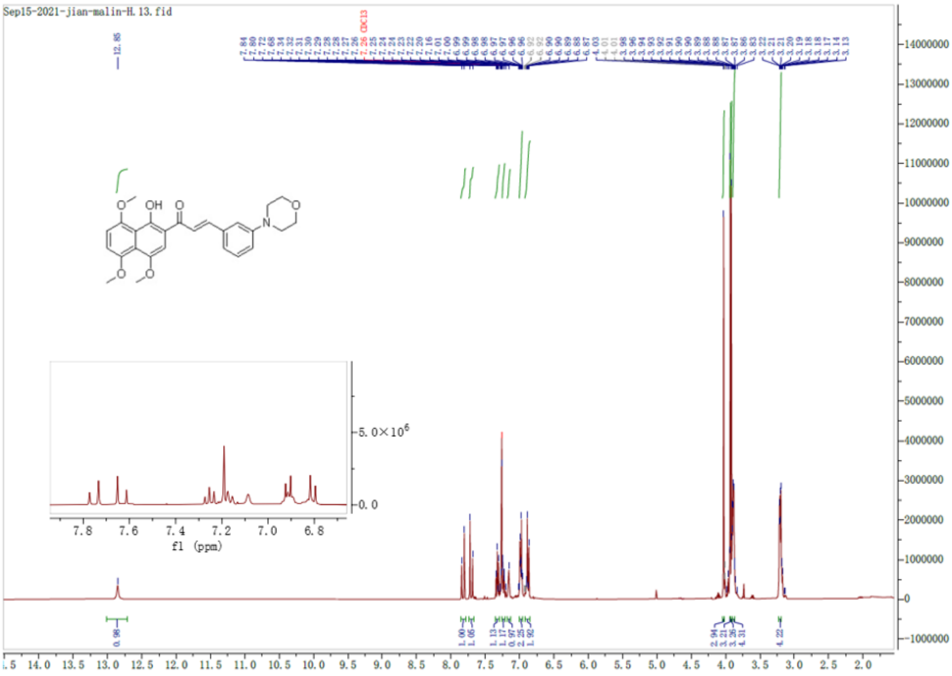

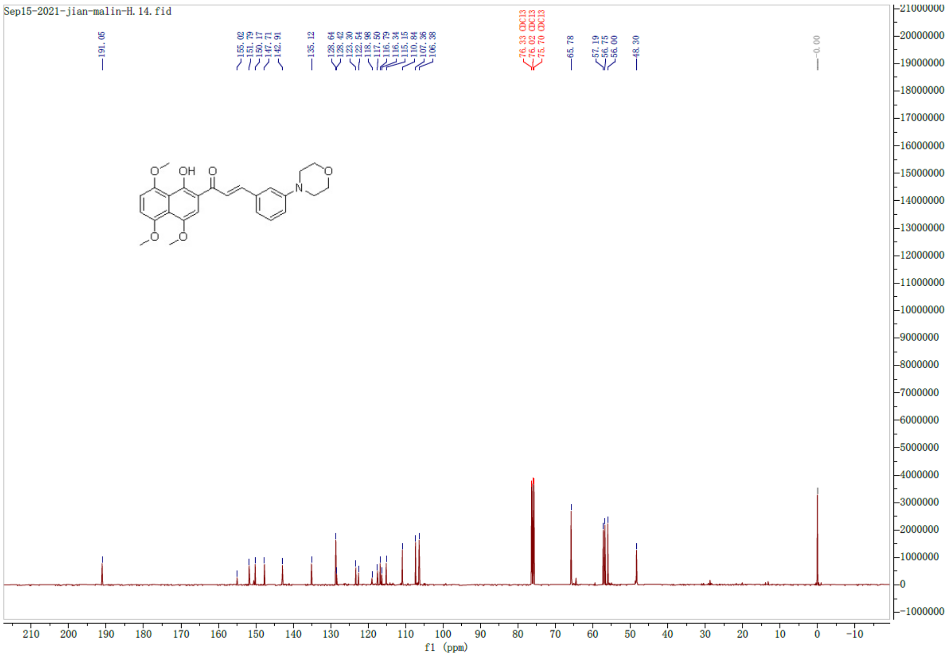

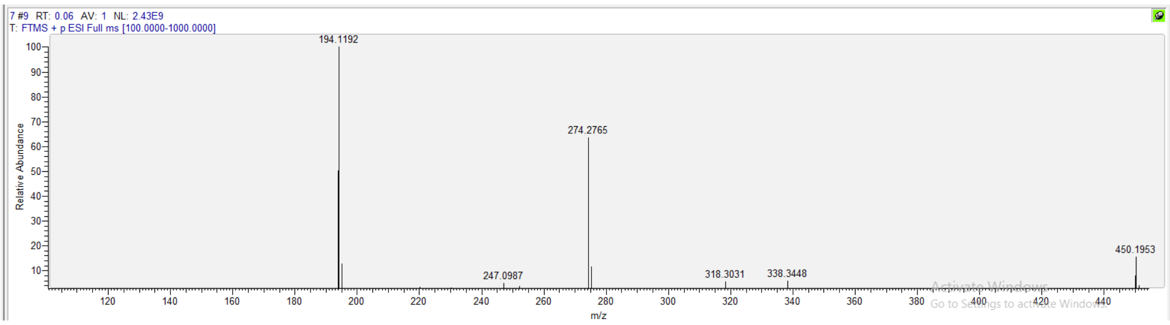

(*E*)-1-(1-hydroxy-4,5,8-trimethoxynaphthalen-2-yl)-3-(2-morpholinophenyl)prop-2-en-1-one (**9g**)

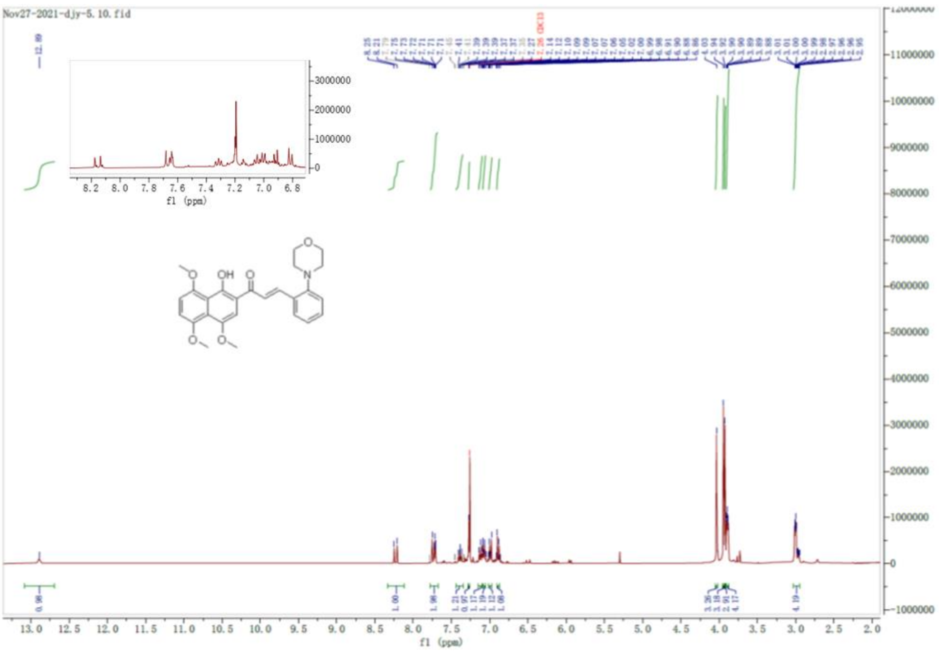

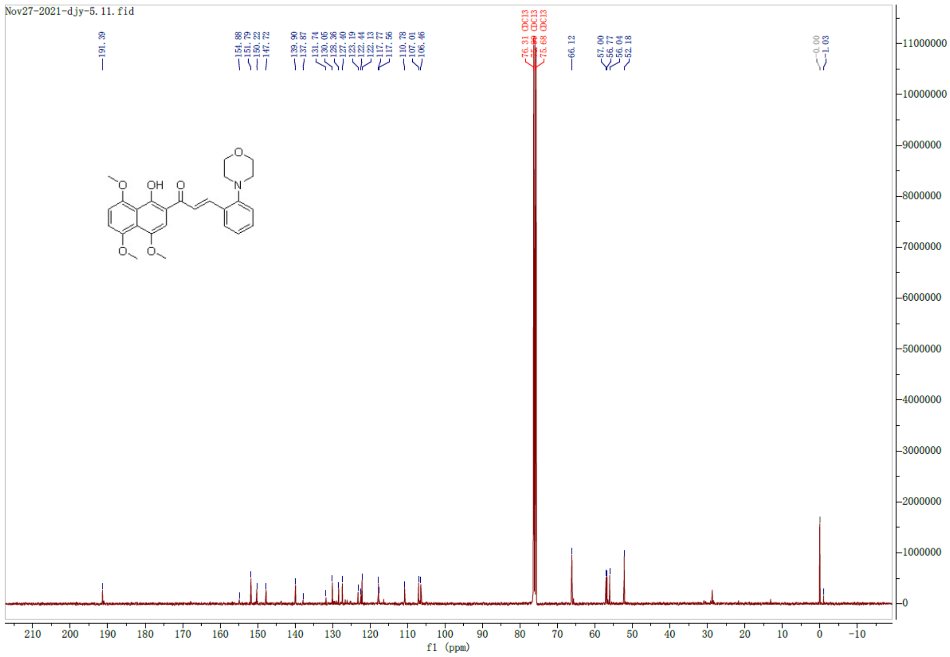

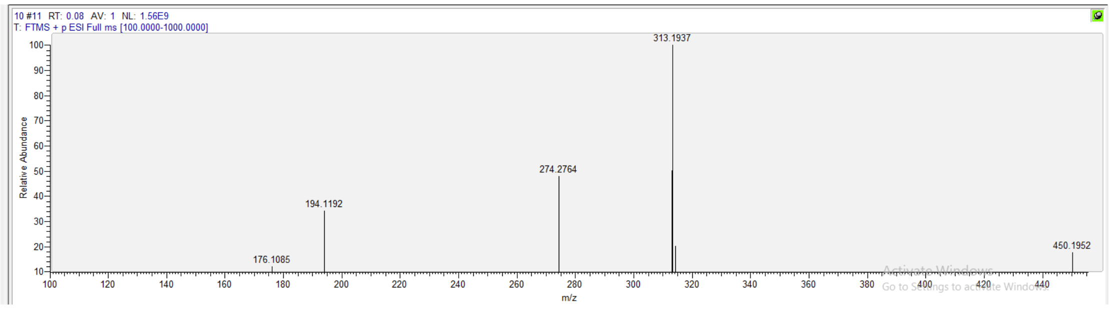

(*E*)-1-(1-hydroxy-4,5,8-trimethoxynaphthalen-2-yl)-3-(2-(4-methylpiperazin-1-yl)phenyl)prop-2-en-1-one (**9h**)

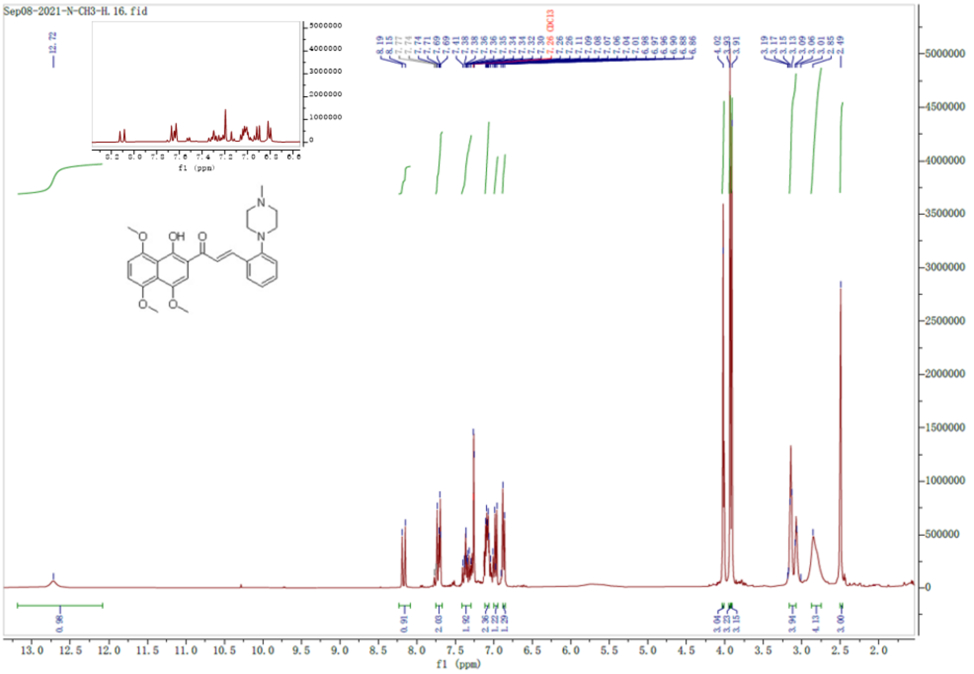

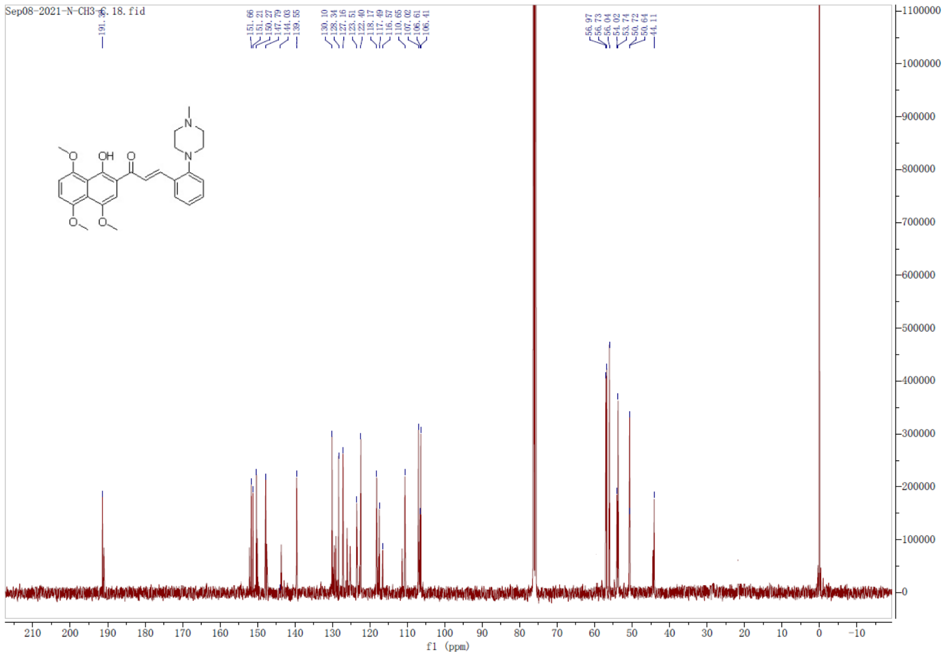

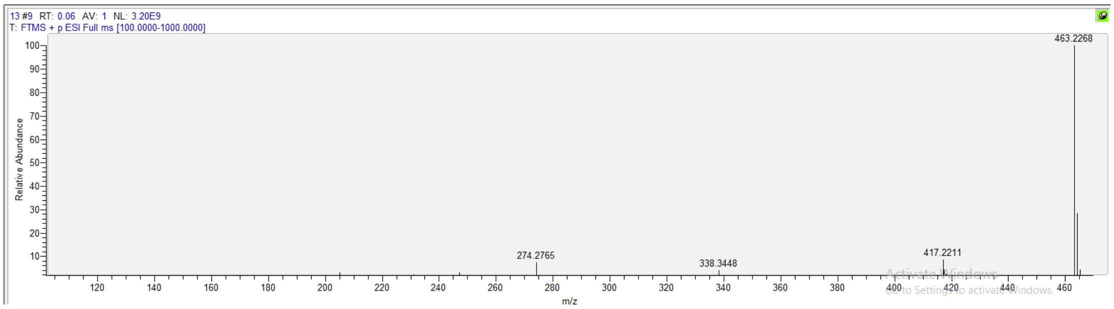

(*E*)-3-(4-(1,1-dioxidothiomorpholino)phenyl)-1-(1-hydroxy-4,5,8-trimethoxynaphthalen-2-yl)prop-2-en-1-one (**9i**)

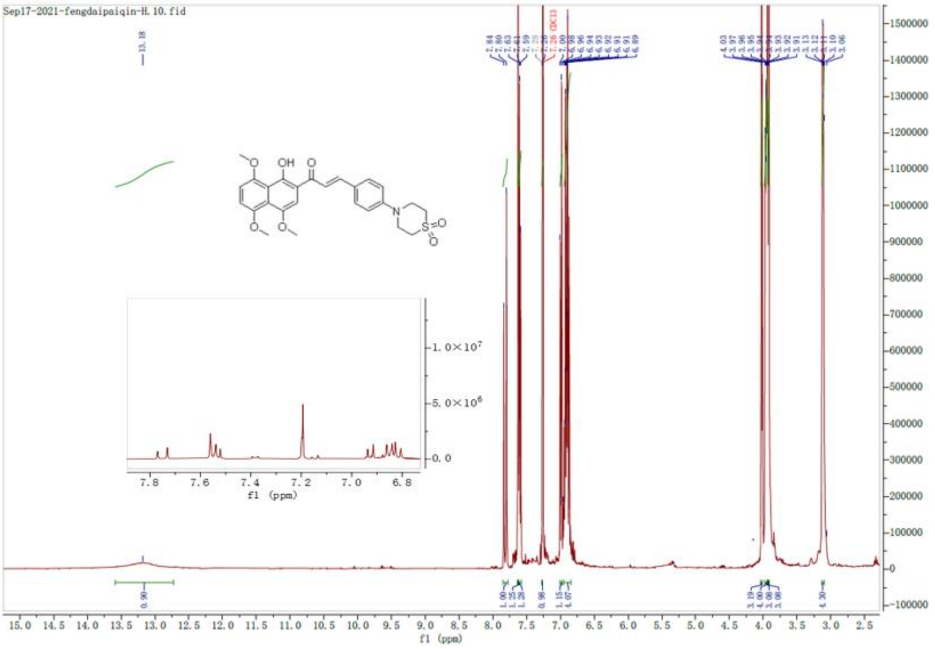

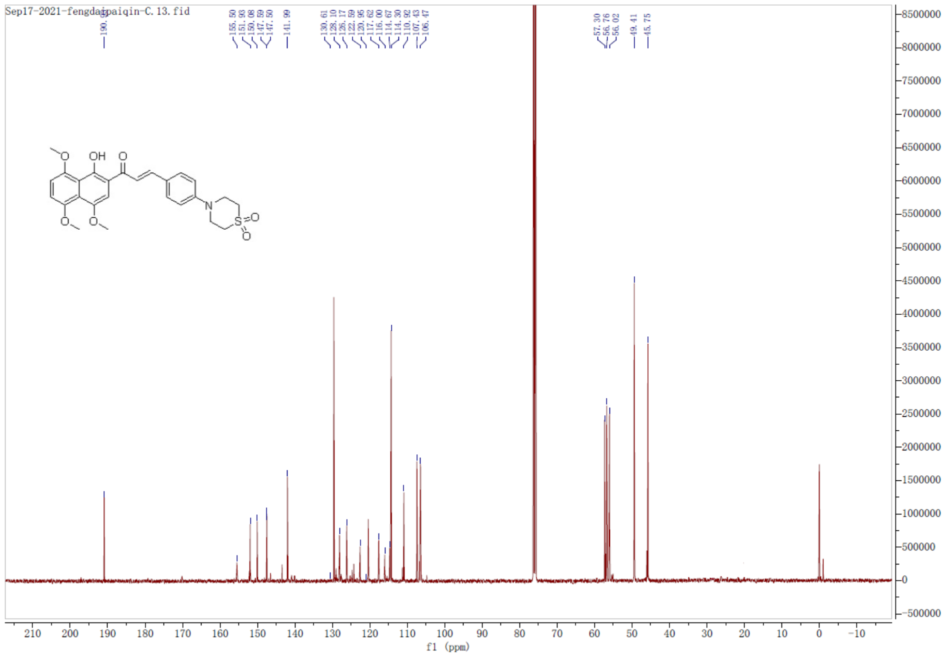

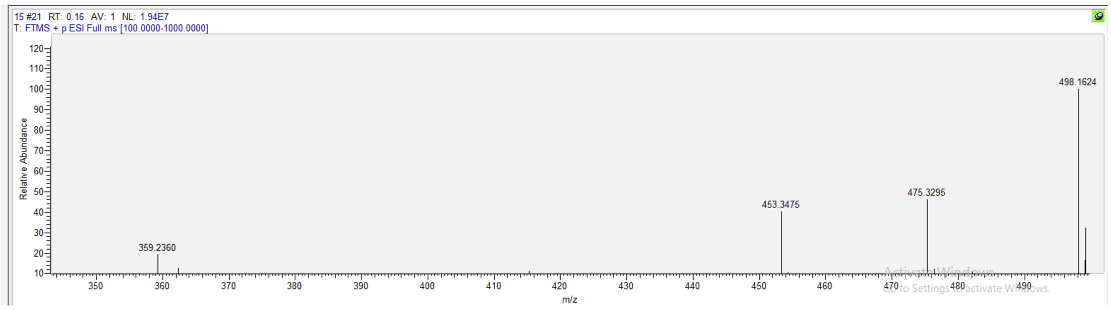

(*E*)-1-(1-hydroxy-4,5,8-trimethoxynaphthalen-2-yl)-3-(4-(pyridin-4-yl)phenyl)prop-2-en-1-one (**9j**)

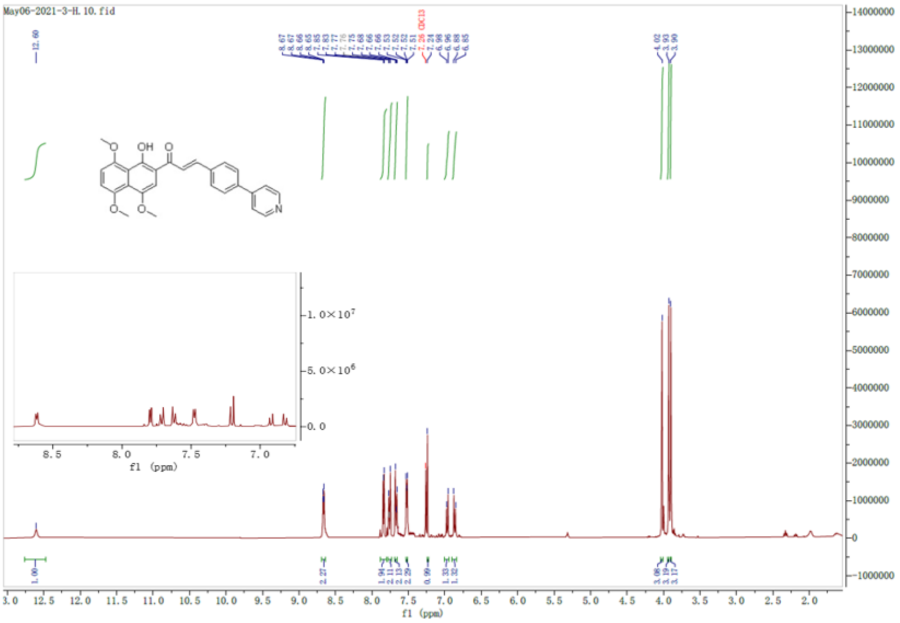

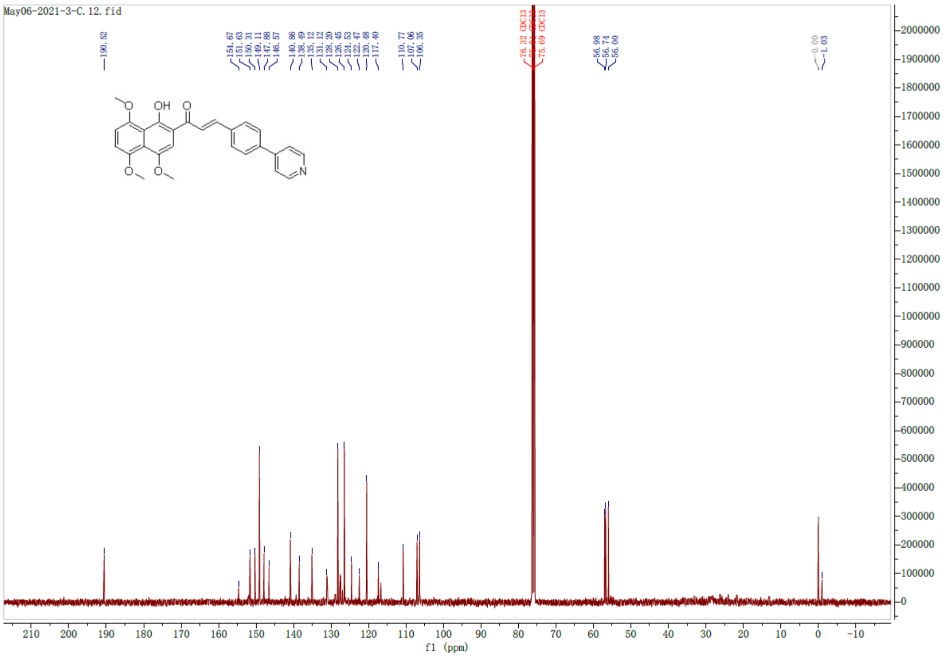

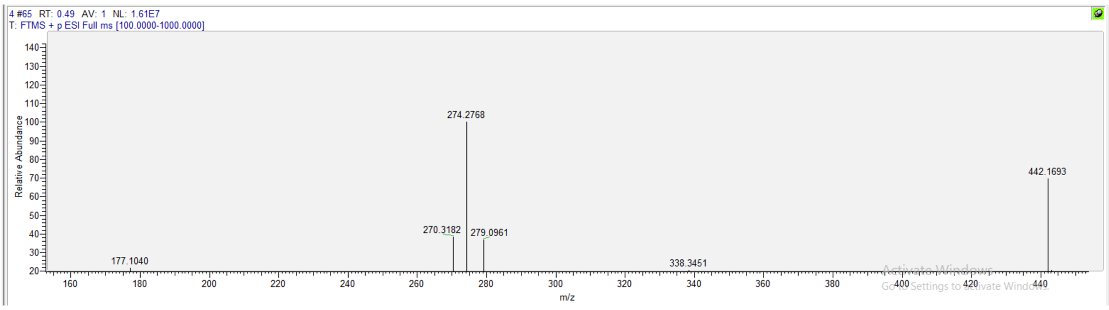

(*E*)-1-(1-hydroxy-4,5,8-trimethoxynaphthalen-2-yl)-3-(3-(pyridin-4-yl)phenyl)prop-2-en-1-one (**9k**)

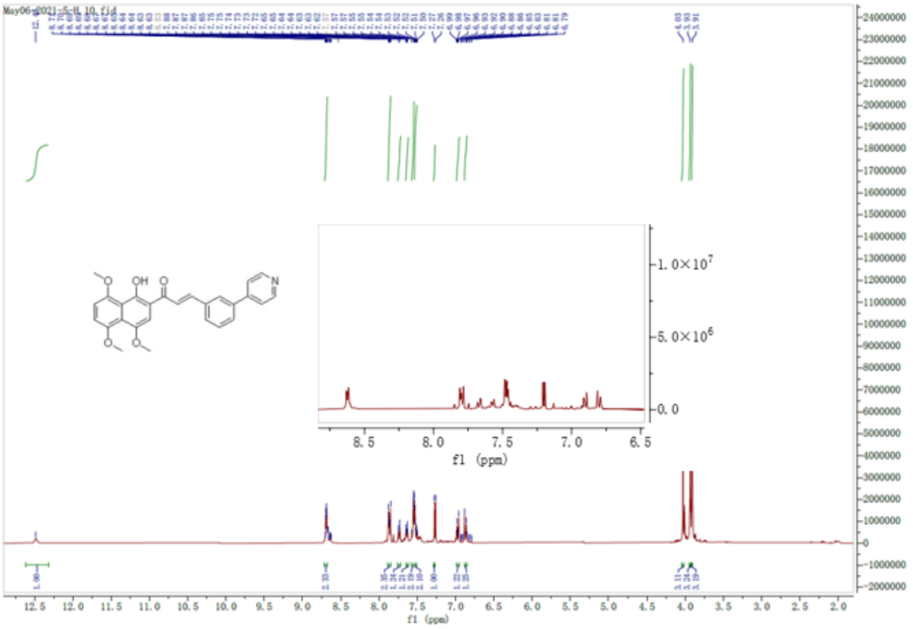

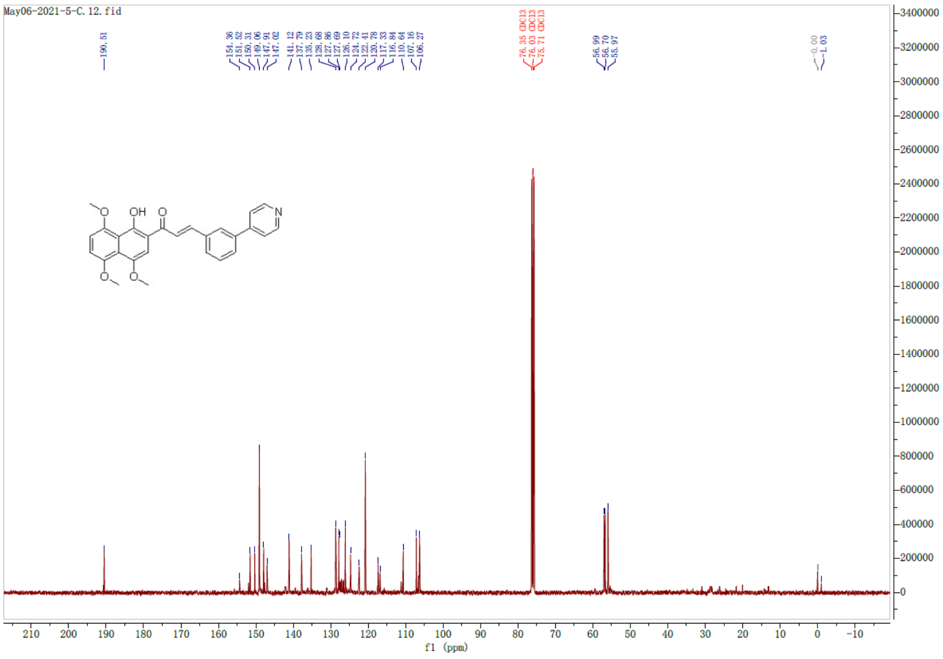

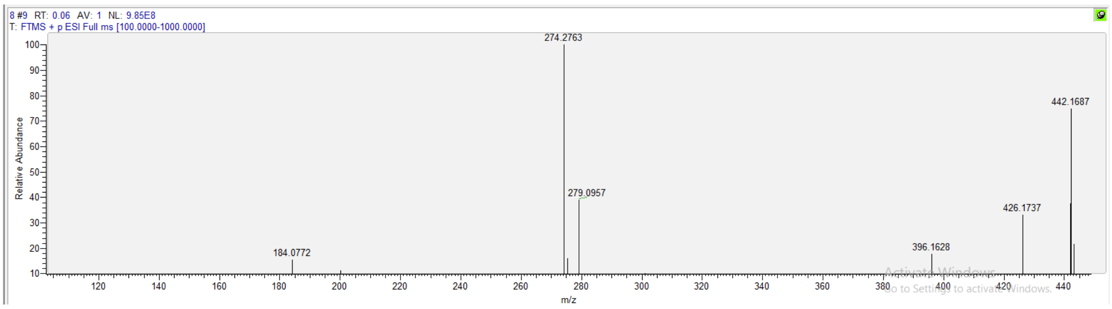

(*E*)-1-(1-hydroxy-4,5,8-trimethoxynaphthalen-2-yl)-3-(2-(pyridin-4-yl)phenyl)prop-2-en-1-one (**9l**)

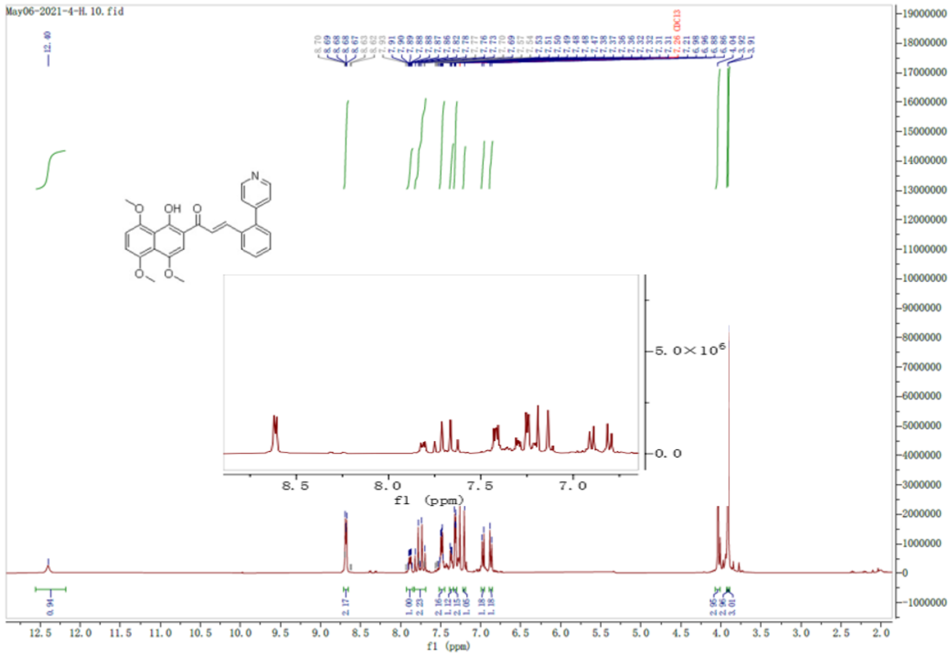

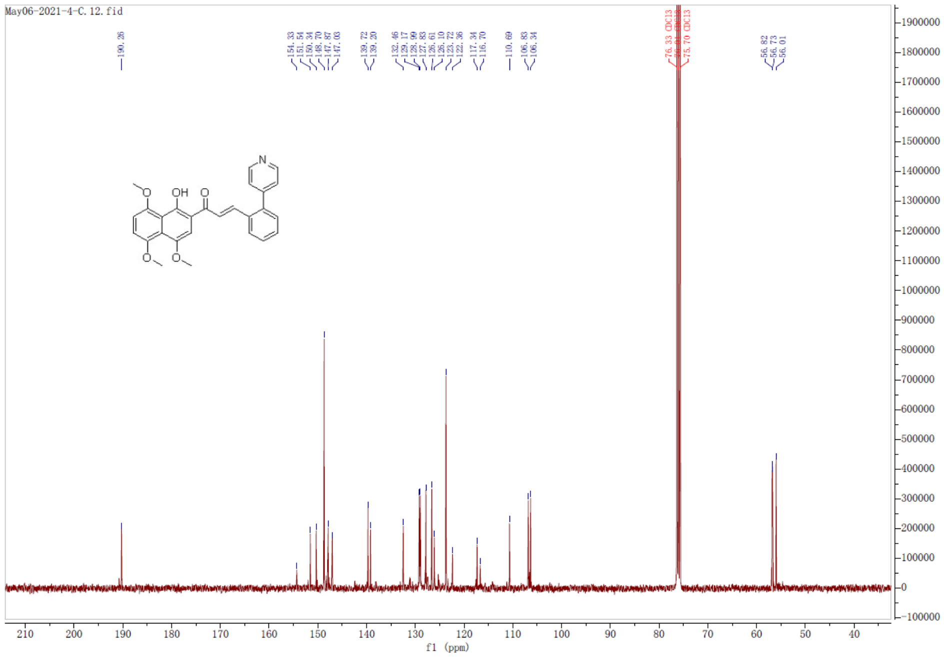

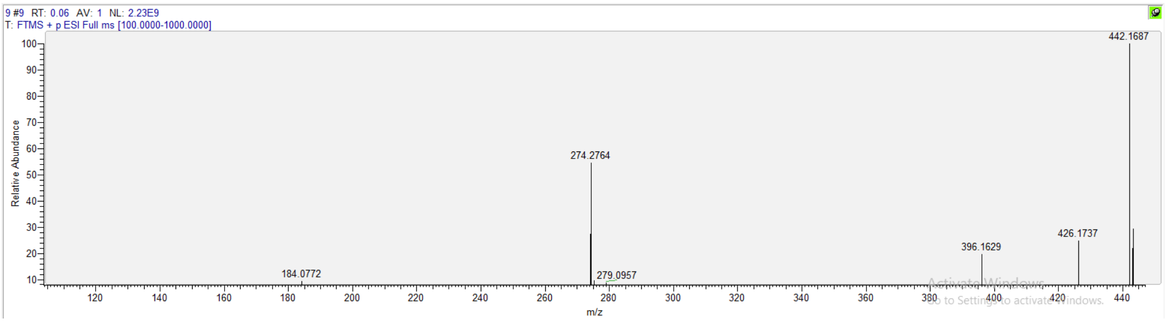

(*E*)-1-(1-hydroxy-4,5,8-trimethoxynaphthalen-2-yl)-3-(pyridin-2-yl)prop-2-en-1-one (**9m**)

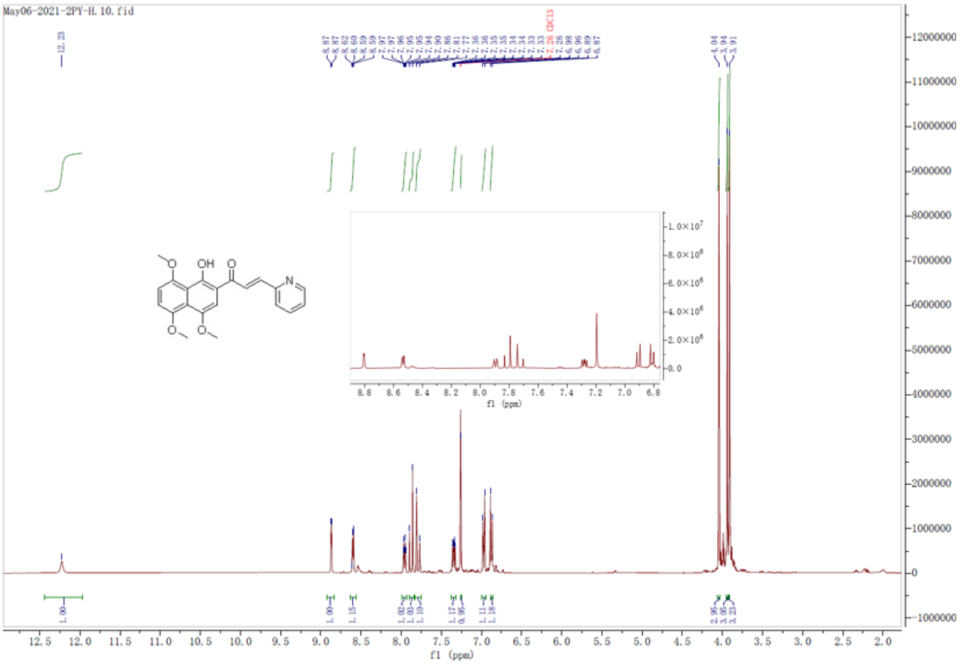

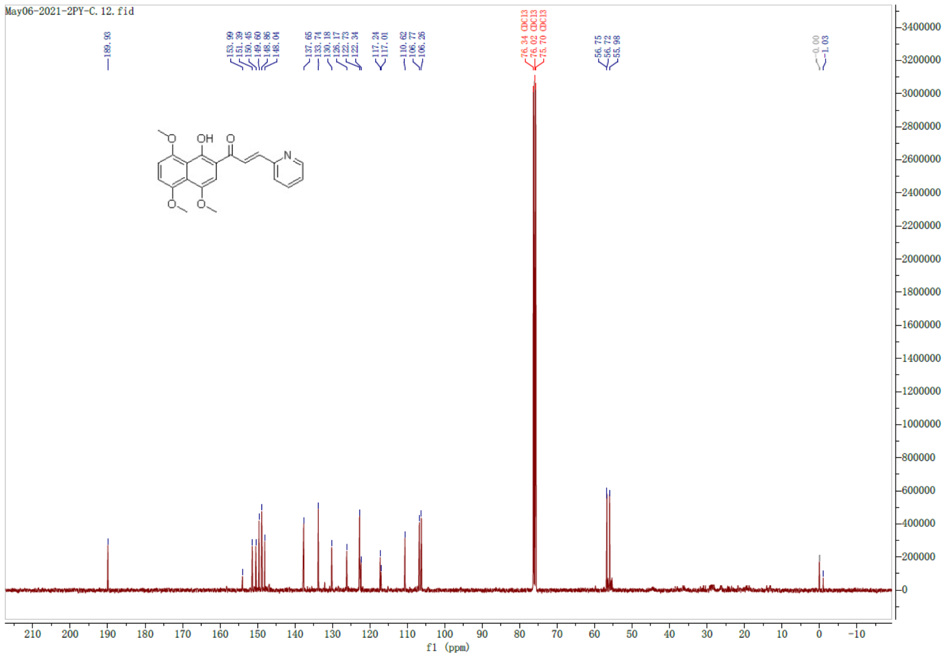

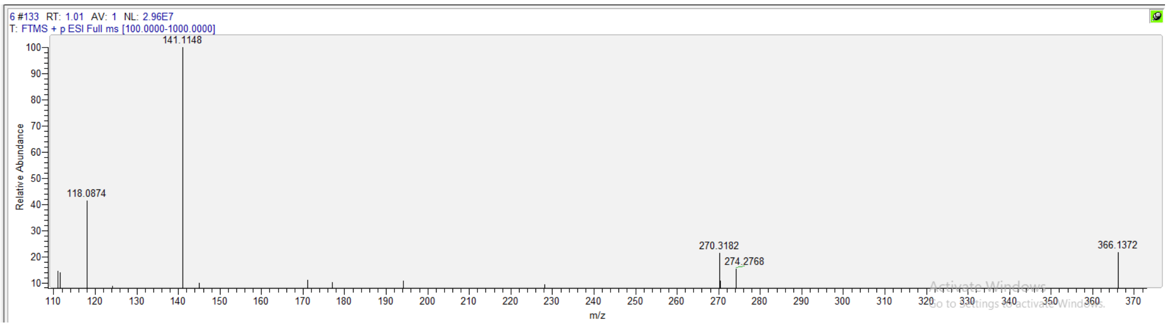

(*E*)-1-(1-hydroxy-4,5,8-trimethoxynaphthalen-2-yl)-3-(5-methoxypyridin-2-yl)prop-2-en-1-one (**9n**)

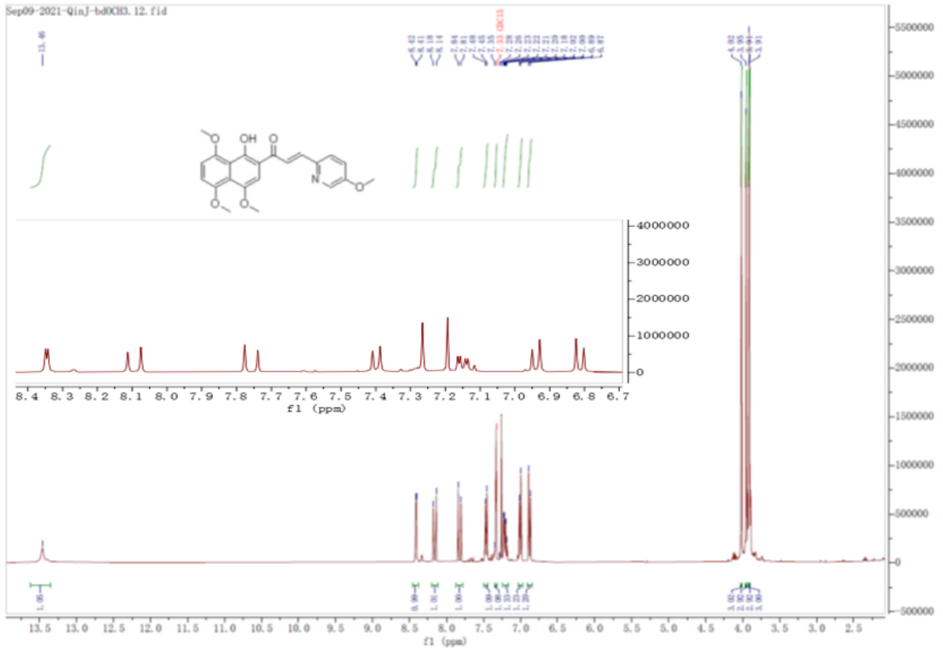

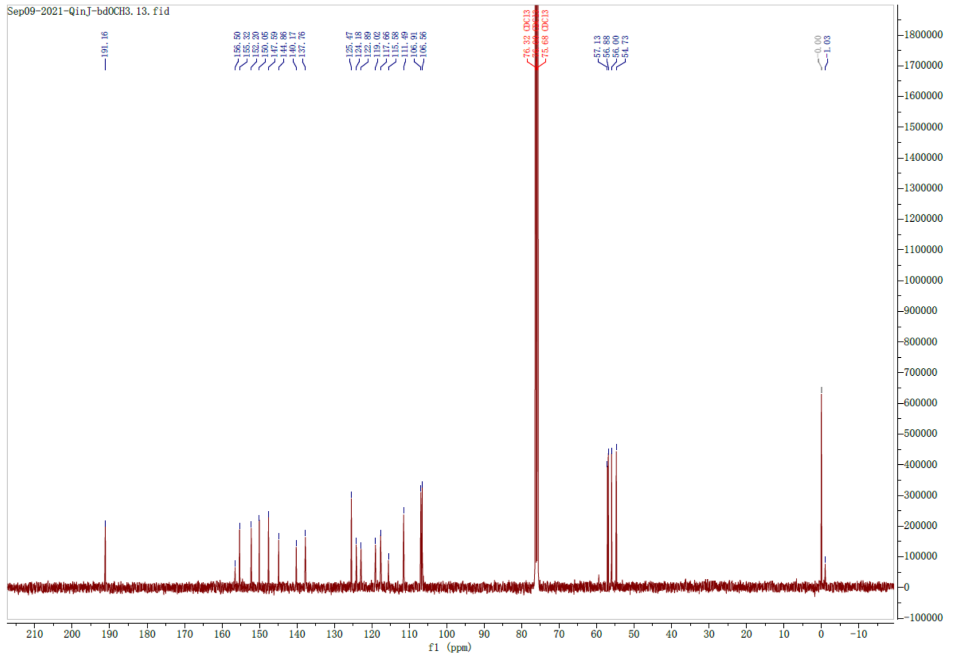

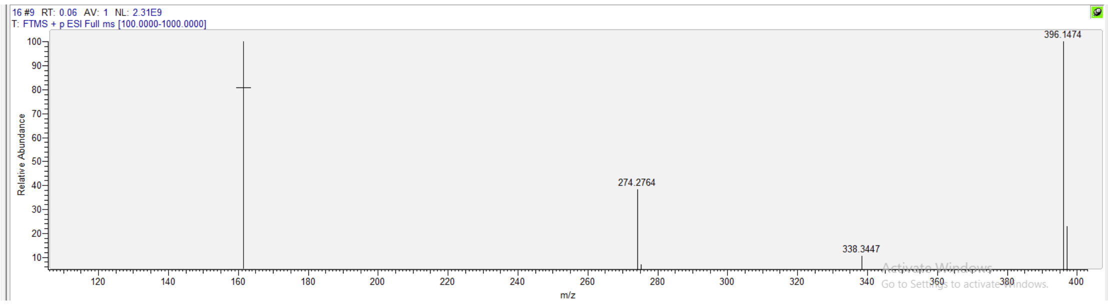

(*E*)-1-(1-hydroxy-4,5,8-trimethoxynaphthalen-2-yl)-3-(quinolin-6-yl)prop-2-en-1-one (**9o**)

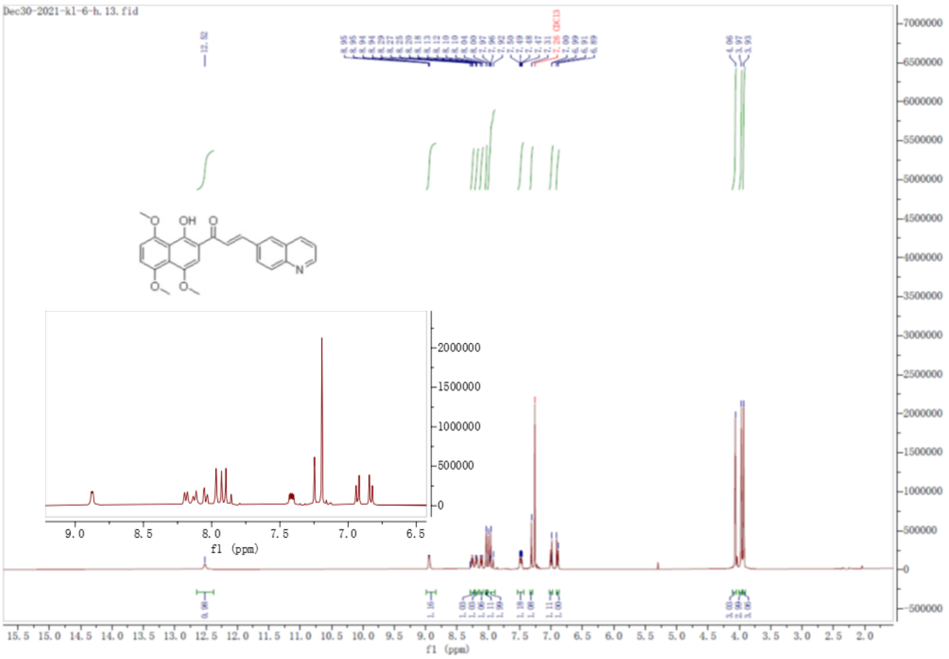

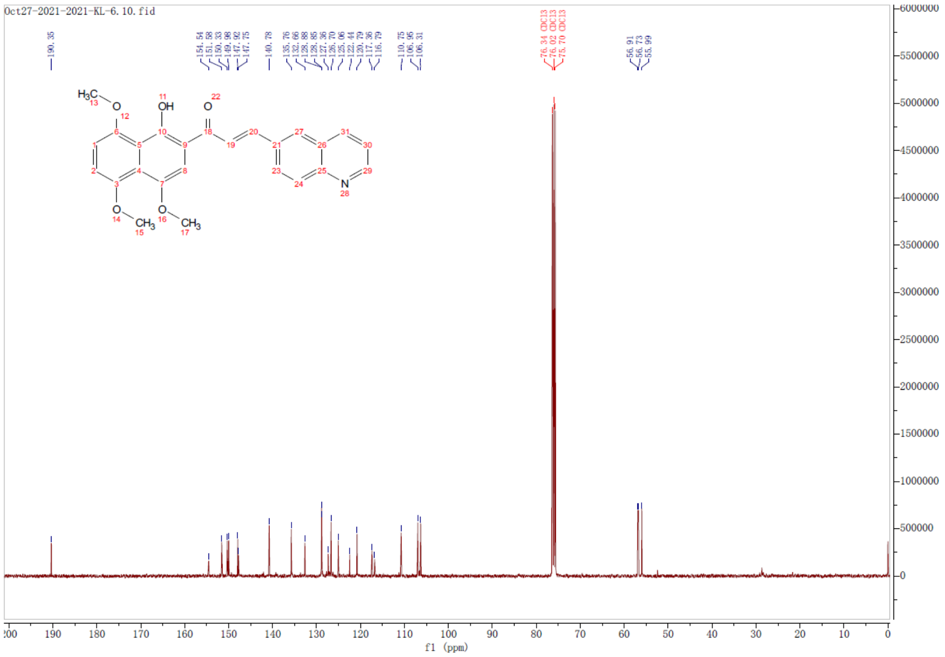

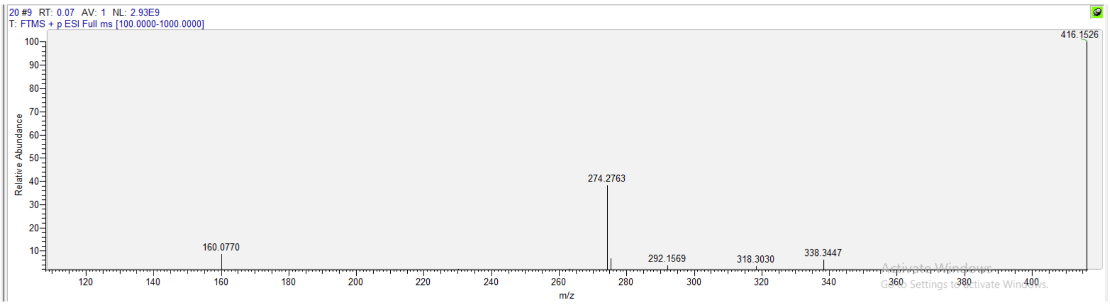

(*E*)-1-(1-hydroxy-4,5,8-trimethoxynaphthalen-2-yl)-3-(quinolin-8-yl)prop-2-en-1-one (**9p**)

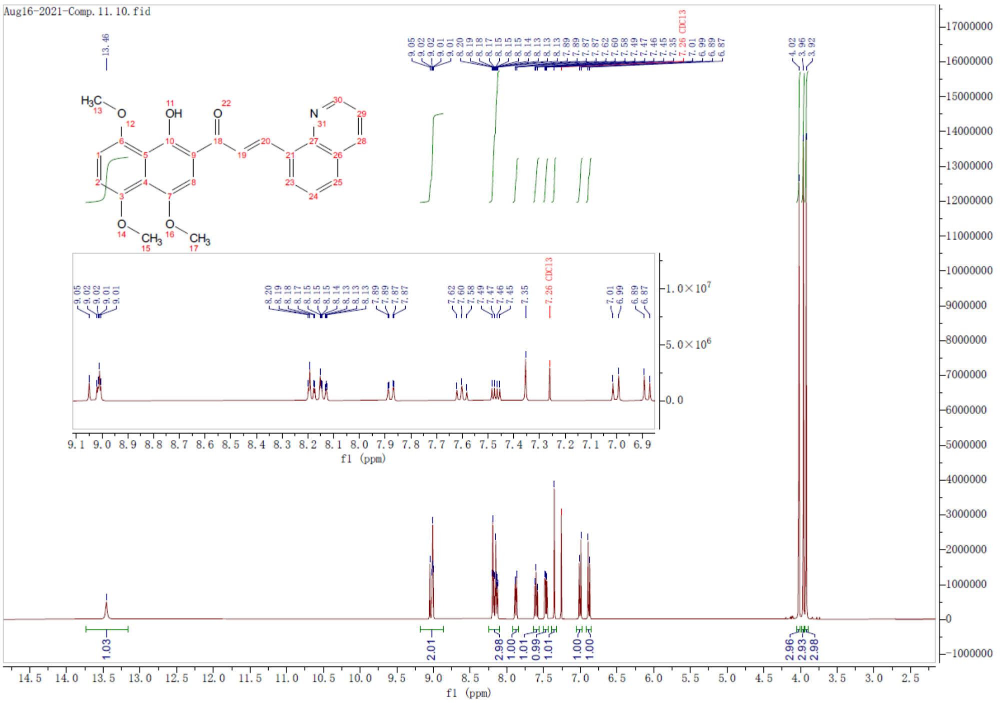

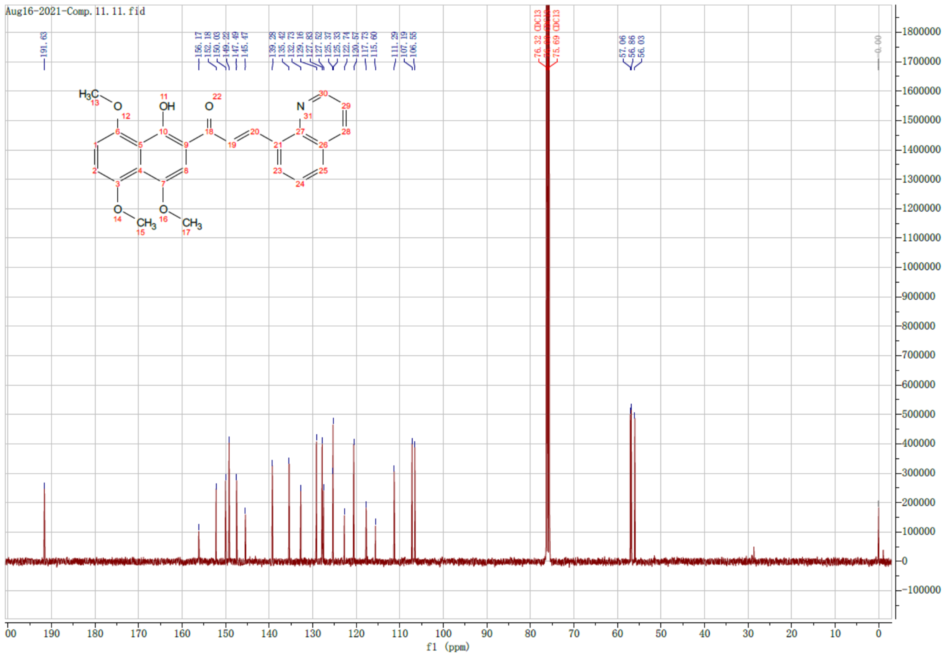

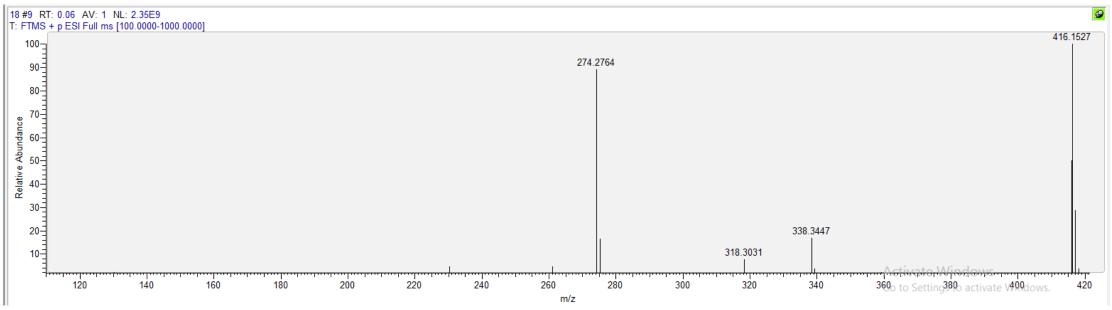

(*E*)-1-(1-hydroxy-4,5,8-trimethoxynaphthalen-2-yl)-3-(quinolin-7-yl)prop-2-en-1-one (**9q**)

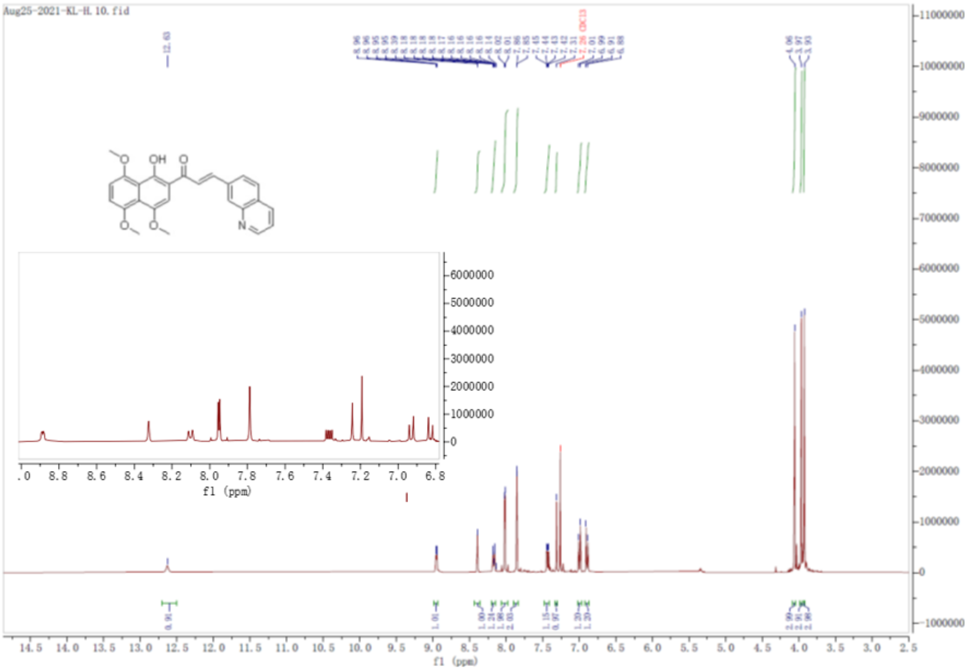

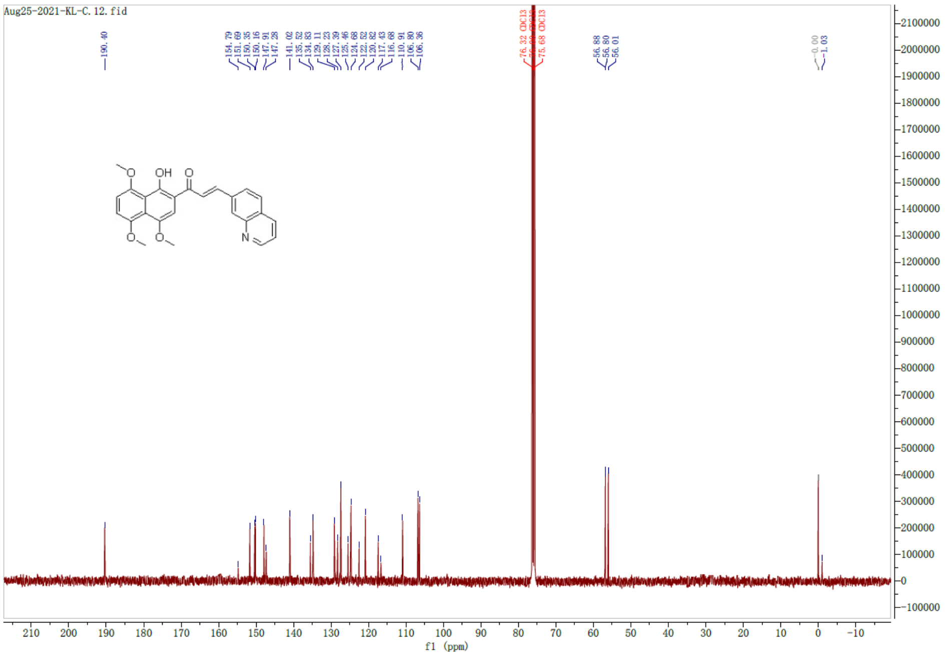

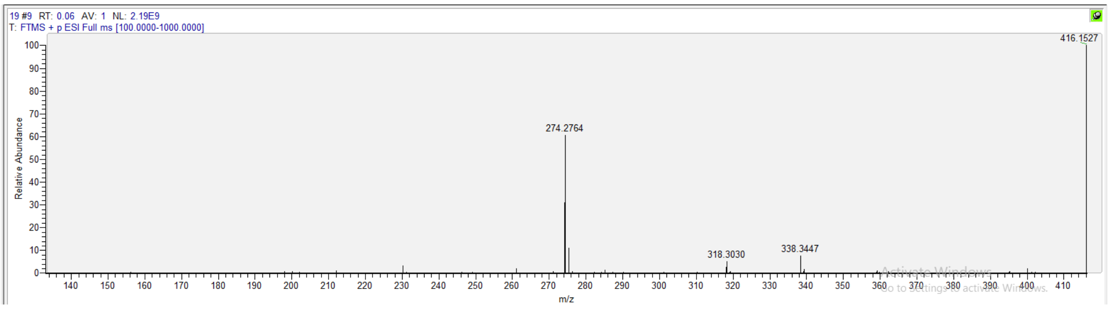

(*E*)-1-(1-hydroxy-4,5,8-trimethoxynaphthalen-2-yl)-3-(quinolin-3-yl)prop-2-en-1-one (**9r**)

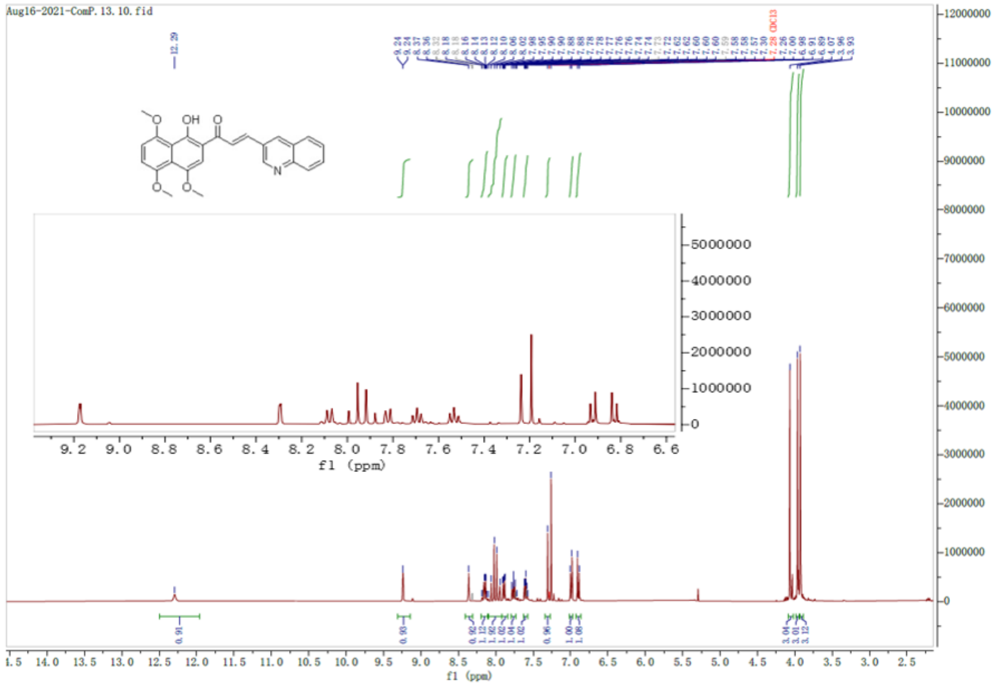

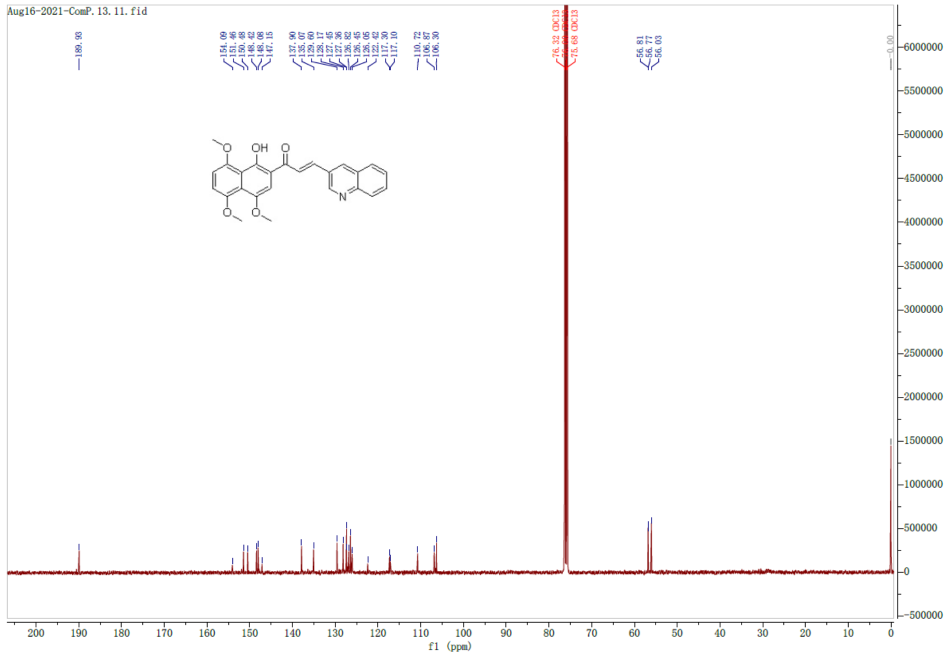

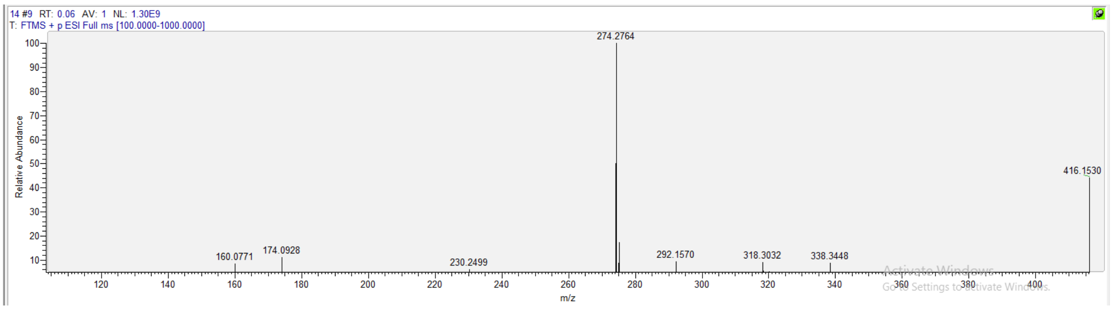

(*E*)-1-(1-hydroxy-4,5,8-trimethoxynaphthalen-2-yl)-3-(quinolin-5-yl)prop-2-en-1-one (**9s**)

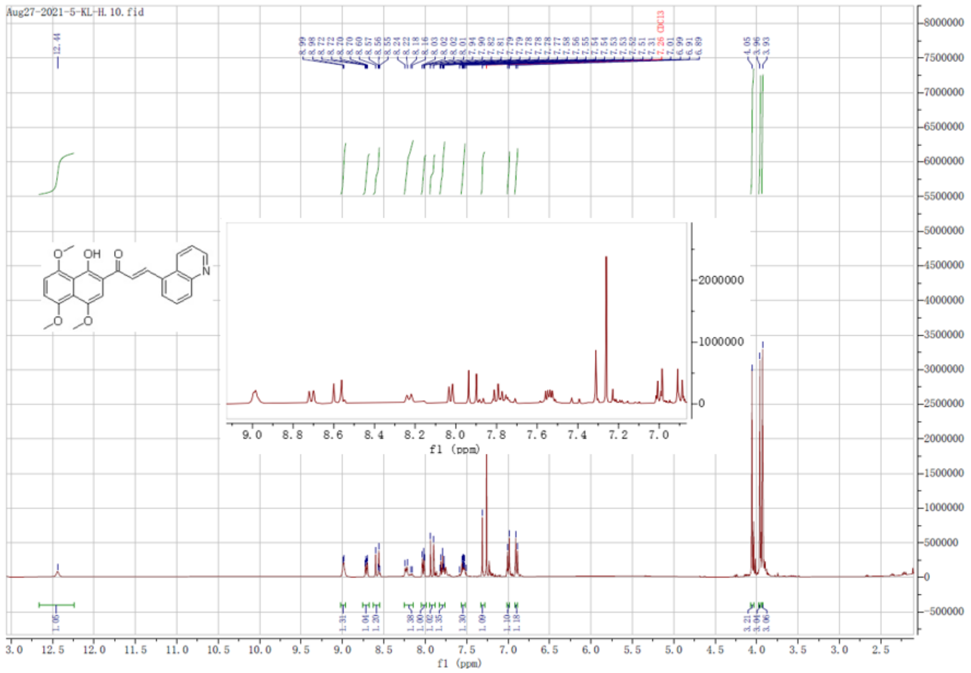

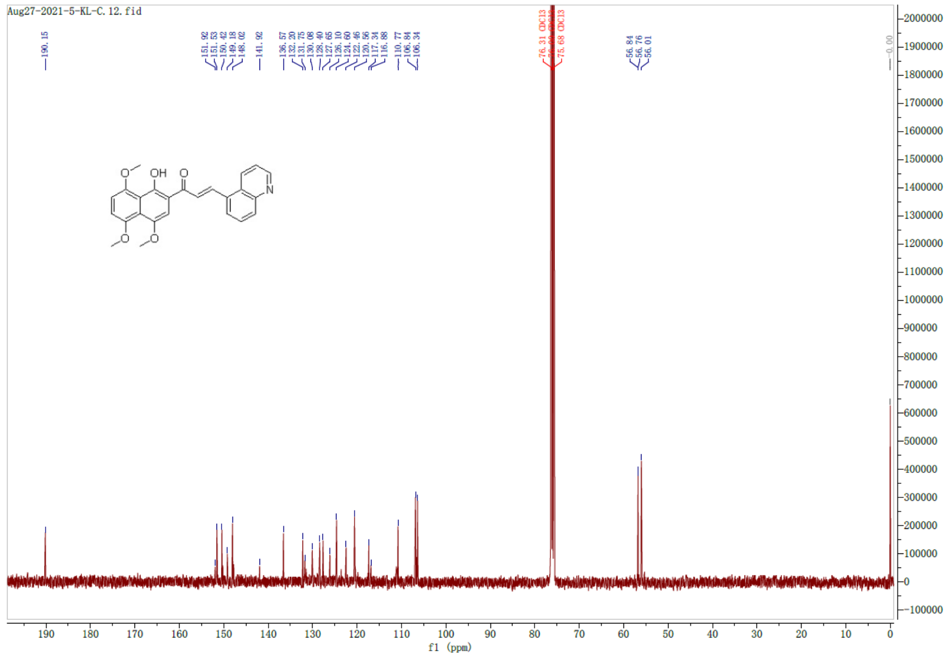

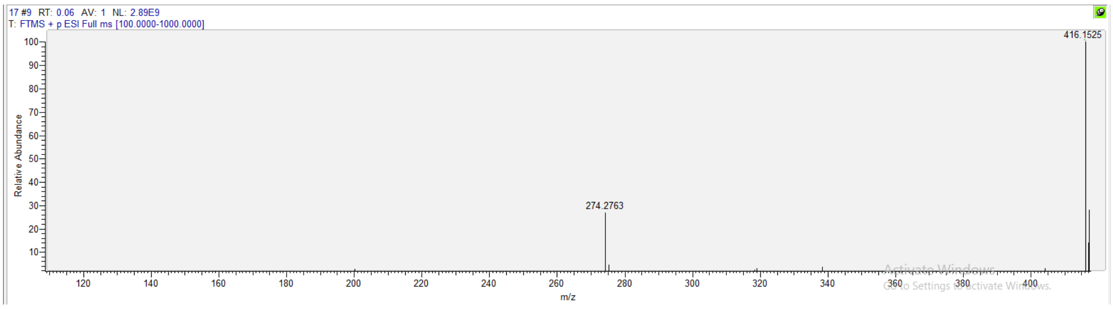

(*E*)-1-(1-hydroxy-4,5,8-trimethoxynaphthalen-2-yl)-3-(isoquinolin-5-yl)prop-2-en-1-one (**9t**)

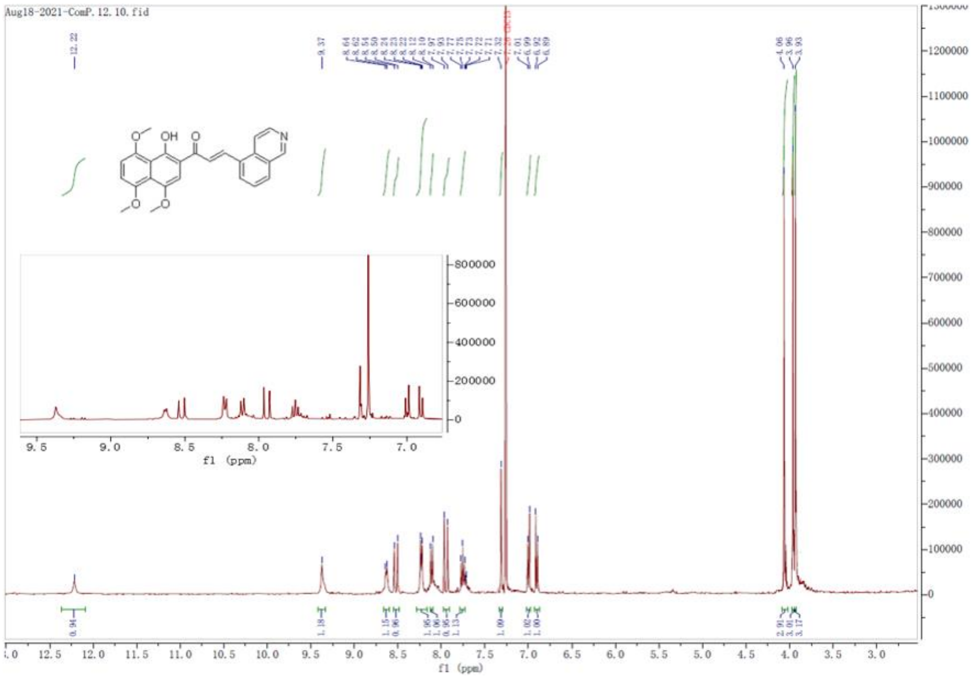

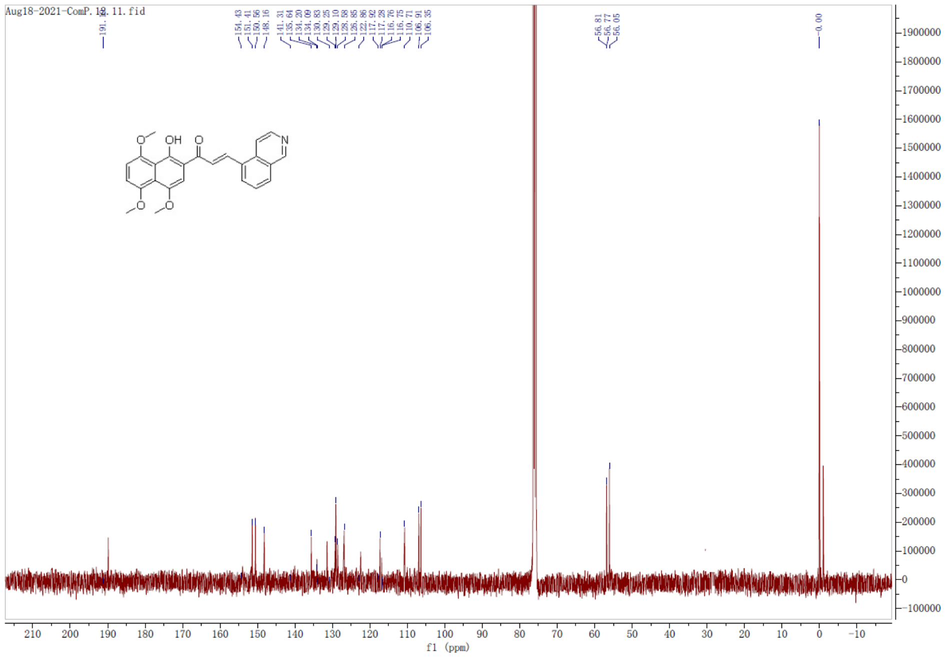

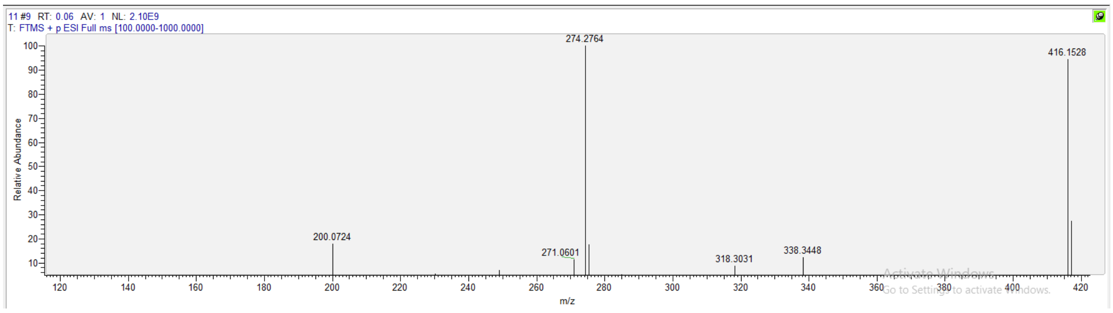

Supplement: Supplemental Material [file IENZ_A_2100366_SM6351.pdf]
